# Evaluation of *Angelica decursiva* reference genes under various stimuli for RT-qPCR data normalization

**DOI:** 10.1038/s41598-021-98434-6

**Published:** 2021-09-23

**Authors:** Yuedong He, Yuan Zhong, Zhenzhen Bao, Weiqi Wang, Xiaoqing Xu, Yanan Gai, Jie Wu

**Affiliations:** 1 School of Pharmacy, Jiangsu Health Vocational College, Nanjing, Jiangsu China; 2 College of Horticulture, Hunan Agricultural University, Changsha, Hunan China; 3 Institute of Botany, Jiangsu Province and Chinese Academy of Sciences, Nanjing, Jiangsu China

**Keywords:** Plant molecular biology, Biotechnology

## Abstract

*Angelica decursiva* is one of the lending traditional Chinese medicinal plants producing coumarins. Notably, several studies have focused on the biosynthesis and not the RT-qPCR (quantitative real-time reverse transcription polymerase chain reaction) study of coumarins. This RT-qPCR technique has been extensively used to investigate gene expression levels in plants and the selection of reference genes which plays a crucial role in standardizing the data form the RT-qPCR analysis. In our study, 11 candidate reference genes were selected from the existing transcriptome data of *Angelica decursiva*. Here, four different types of statistical algorithms (geNorm, NormFinder, BestKeeper, and Delta Ct) were used to calculate and evaluate the stability of gene expression under different external treatments. Subsequently, RefFinder analysis was used to determine the geometric average of each candidate gene ranking, and to perform comprehensive index ranking. The obtained results showed that among all the 11 candidate reference genes, SAND family protein (*SAND*), protein phosphatase 2A gene (*PP2A*), and polypyrimidine tract-binding protein (*PTBP*) were the most stable reference genes, where Nuclear cap binding protein 2 (*NCBP2*), TIP41-like protein (*TIP41*), and Beta-6-tubulin (*TUBA*) were the least stable genes. To the best of our knowledge, this work is the first to evaluate the stability of reference genes in the *Angelica decursiva* which has provided an important foundation on the use of RT-qPCR for an accurate and far-reaching gene expression analysis in this medicinal plant.

## Introduction

*Angelica decursiva* (Miq.) Franch. et Sav. is an important traditional medicinal plant form the genus *Angelica*, family Umbelliferae. The major bioactive components of this herbal drug are coumarins and this drug has been listed as one of the main special coumarin sources by the Chinese Pharmacopoeia. Coumarin has a core structure (2H-1-benzopyran-2-one core), that widely exists in higher plant species like the Rutaceae, Leguminosae, and Umbelliferae. Besides, they are slightly distributed in both animals and microorganisms^1^. Recent pharmacological studies have proved that the 70% ethanol extract of *A. decursiva* has an anti-inflammatory activity^2,3^, antioxidant^4,5^, inhibitory effect on C6 rat glioma cells^6^ and inhibits the proliferation of osteogenic sarcoma cells^7^. Hence, certifying the supply of coumarin compounds from the drug has been a long-term practice^8^. Conversely, there is a huge shortage of coumarin raw materials, which has been attributed to low abundance, season, or region-dependent sourcing. Also, because of its complicated structure and multiple chiral centers^9^, there lacks a feasible method that has the capacity of mass chemical synthesis. Therefore, genetic and metabolic engineering strategies used in the production of coumarin compounds have become the main developing research direction. Subsequently, the study of functional genes that are present in their biosynthetic pathways is a study of interest.

In recent years, the rapid development of next-generation sequencing (NGS) technologies such as Roche / 454 and Illumina HiSeq platforms, has made it possible to study the distribution of mRNA and their expression levels in different tissues and cells. Currently, due to its high sensitivity, accurate quantification, and strong specificity, the RT-qPCR technology has become one of the most powerful tools for studying gene transcription levels. Besides, it quantifies the relative abundance and improves the quantitative accuracy of target genes^10–12^. Nonetheless, the accuracy of this technique is affected by several factors^13^, like the amplification efficiency of the target gene, the RNA yield, quality, and the reverse transcription efficiency that is present in different samples. Therefore, to overcome experimental errors and assure accuracy of experimental results, a certain number of internal reference genes, also called housekeeping genes, are often selected for calibration and standardization^14,15^. There genes are usually used as a reference for either tissues or cells when they are stably expressed in a diversity of experimental conditions, and when changes are detected in the expression level of a target gene. Among the most commonly used internal reference genes for RT-qPCR are Glyceraldehyde-3-phosphate dehydrogenase (*GAPDH*), Elongation factor-1α (*EF-1α*), Tubulin (*TUB*), β-Actin (*ACT*), 18S ribosomal RNA protein (*18S*), Polyubiquitin 10 (*UBQ10*), etc. These housekeeping genes play a significant role in the cell structure maintenance and during primary metabolic activities. Moreover, more internal reference genes have been identified from the gene expression chip data. They include protein phosphatase 2A (*PP2A*), SAND family protein (*SAND*), Tap42-interacting protein of 41 kDa (*TIP41*), etc. Consequently, the reference gene screening and evaluation have been accomplished for most species, such as rice^16^, *Lycoris aurea*^17^, radish^18^, potato^19^, *Peucedanum praeruptorum*^20^, etc. However, no study has demonstrated a systematic selection of reference genes in *A. decursiva* under external challenges (abiotic stress and hormone treatments). The study of the coumarin biosynthesis pathway is one of our primary research interests relates. Here, previous studies have characterized some candidate genes that associate with *A. decursiva*^8^. However, it is imperative to find suitable reference genes, that enhance the detection of differential gene expression levels under various experimental conditions in *A. decursiva* using the RT-qPCR application.

In our study, 11 candidate reference genes (*SAND*, *PP2A*, *PTBP*, *ACT*, *CYP2*, *EXP-1*, *GAPDH*, *TUBA*, *NCBP2*, *UBQ10*, and *TIP41*) were selected about the transcriptome sequencing datasets of *A. decursiva*. Besides, 7 different forms of treatments such as cold (4 °C), drought (20% PEG 6000), methyl jasmonate (25 mM MeJA), salt (600 mM NaCl), oxidative (50 mM H_2_O_2_), ultraviolet (UV) induction, and metal (500 mM CuSO_4_) were set. Next, the application of four statistical algorithms (geNorm, NormFinder, BestKeeper, and Delta Ct) to evaluate their expression stability for normalization, and comprehensive stability ranking was also performed by RefFinder. In conclusion, our work provides a basis for further studies on gene expression profiling and the regulation mechanisms of coumarin biosynthesis in *A. decursiva* under diverse experimental conditions.

## Results

### Selection of candidate reference genes, evaluation of amplification specificity and PCR efficiency

Here, 11 candidate reference genes were selected based on the transcriptome data of *A. decursiva* (unpublished). Table 1 lists all the candidate reference gene names and abbreviations, homologous genes from Arabidopsis, primer sequences, amplification length, annealing temperature (°C), PCR efficiency (E), and correlation coefficient (R^2^). Next, conventional PCR and RT-qPCR were used to verify the primer-specific amplification of all candidate reference genes. As illustrated in Fig. S1, based on agarose gel electrophoresis and melting curve analysis which showed a peak, the 11 primer pairs were amplified by a single specific amplicon. Here, the amplification efficiency range of *CYP2* and *PP2A* was 1.711 and 1.880 respectively whereas the correlation coefficient range of *UBQ10* was 0.888, and that of *PP2A*, *PTBP*, *TIP41*, and *ACT* was 1.000.

**Table 1:** Details of primers used in this study.

Gene abbreviation	Gene name	Arabidopsis homolog locus	Primer sequences (5′–3′) (forward/reverse)	length	Tm (°C)	PCR efficiency	R^2^
*CYP2*	Cyclophilin 2	AT4G33060	CGCCACTTTTTTTGTTCTCT/TTGCGGATTATATTCCGACA	108	82.9	1.711	0.971
*EXP-1*	S-adenosyl-l-methionine-dependent methyltransferases superfamily protein	AT2G32170	CCAAGTAGGAGCTTGGGATG/CACACCACCGTCCTTTAGAA	109	81.7	1.789	0.958
*GAPDH*	Glyceraldehyde 3-phosphate dehydrogenase	AT1G42970	TGGTTCCCTTAACGATACCA/CTTACGTTGTTGGCGTGAAT	138	82.1	1.713	0.999
*NCBP2*	Nuclear cap binding protein 2	AT5G44200	TAGATGGCATGACAGGTGGA/CAGGTACGCGATGAATATCG	145	85.6	1.333	0.993
*PP2A*	protein phosphatase 2A subunit A3	AT1G13320	GGTTGCCAATCAGCTCTATG/AACTTGGTGACTTTGCCAGC	141	85	1.880	1.000
*PTBP*	Polypyrimidine tract-binding protein	AT3G01150	AATGTAAAGGCCTTCAGCGA/GGAGCAGCATGAGGATTCTG	110	85.2	1.847	1.000
*SAND*	SAND family protein	AT2G28390	GAGCCTCATGAATCCCTCAG/CCCAAGCAAAGGTGTCATAT	126	82.1	1.845	0.972
*TIP41*	TIP41-like family protein	AT4G34270	CACTTGCATCAAAAGAGCCT/GAAGAAACCAACAGCTTGGC	126	82.7	1.838	1.000
*UBQ10*	Polyubiquitin 10	AT4G05320	TTAAGCAGGGATCAAAACCC/TTTGCTGGAAAGCAGCTAGA	124	84.6	1.721	0.888
*ACT*	Actin 2	AT3G18780	TGCTGGTCGTGATCTCACTG/GGTTTCAAGCTCTTGCTCGT	148	85.2	1.850	1.000
*TUBA*	Beta-6-tubulin	AT5G12250	CACCCAGCTTTGGTGATCTC/AAGCCTAGGGAAGGGGATTA	134	86	1.685	0.999

### Expression profile of candidate reference genes

**Figure 1:**
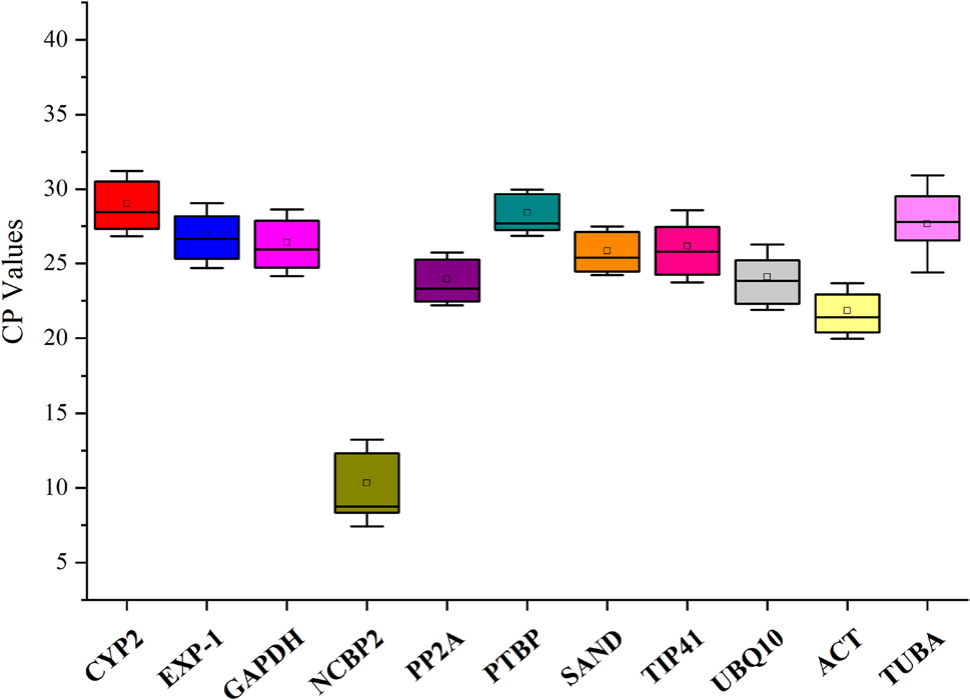
The RT-qPCR Cp values for 11 candidate reference genes in all samples. The expression data is shown as the Cp value of each reference gene in the samples of *A. decursiva*. Boxes indicate the 25th/75th percentiles, the lines represent the median, squares represent the means and whiskers represent the maximum and minimum values.

Here, the cycle threshold values (Cp) showed the number of cycles when the generated fluorescent signal reached a level that could be detected. Therefore, in this study, the expression profile of the candidate reference genes were directly determined using the obtained Cp values. As shown in Fig. 1, the average Cp values of these 11 reference genes are distributed between 10 and 30, and a majority of them are distributed between 23 and 27. Among all the candidate reference genes, *NCBP2* had the lowest average Cp values, whereas *CYP2*, *PTBP*, and *TUBA* had the highest average Cp values. Moreover, each reference gene had a different coefficient of variation under different conditions. Also, it was observed that *SAND* and *PTBP* had the lowest variability, whereas *TUBA*, which had the highest Cp value, had the highest variability. This value ranged between 25 and 32. Therefore, it may not be qualified as a reference gene. Through the Cp value combined with box-plot not only display the expression profile of the reference gene, but also give us a glimpse in their stability (Table S2). However, considering the complexity of their surroundings, we need to check the proper use of the references in every particular experimental condition. Thus, more statistical tools and further analyses are required.

### Expression stability of candidate reference genes

Based on the relative expression levels of the 11 candidate reference genes, four algorithms such as BestKeeper, geNorm, NormFinder, and Delta Ct were used to examine their expression stability. Subsequently, the RefFinder tool was employed to sequence the expression stability of all these candidate reference genes and select the most suitable ones.

**Figure 2:**
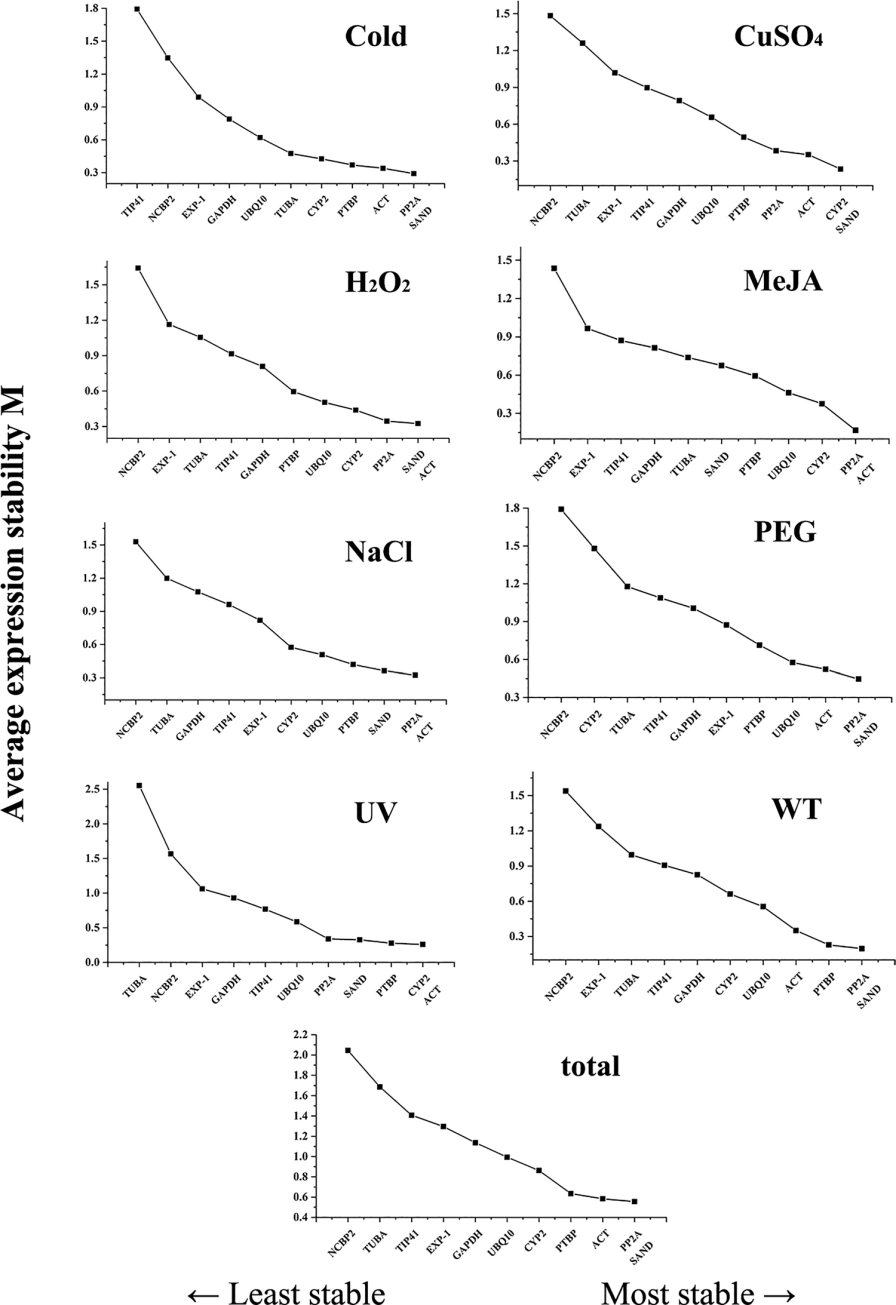
Average expression stability values (M) of the 11 candidate reference genes using geNorm software. Expression stability was evaluated in samples from *A. decursiva* was submitted to cold stress, drought stress (20% PEG), Methyl jasmonate (MeJA) stress, salt stress (0.5 M NaCl), oxidative stress (H_2_O_2_), ultraviolet (UV) induction, Metal stress (CuSO_4_), untreated (WT) and 'total' (all treated samples). The least stable genes are on the left with higher M-value and the most stable genes on the right with lower M-value.

**Figure 3:**
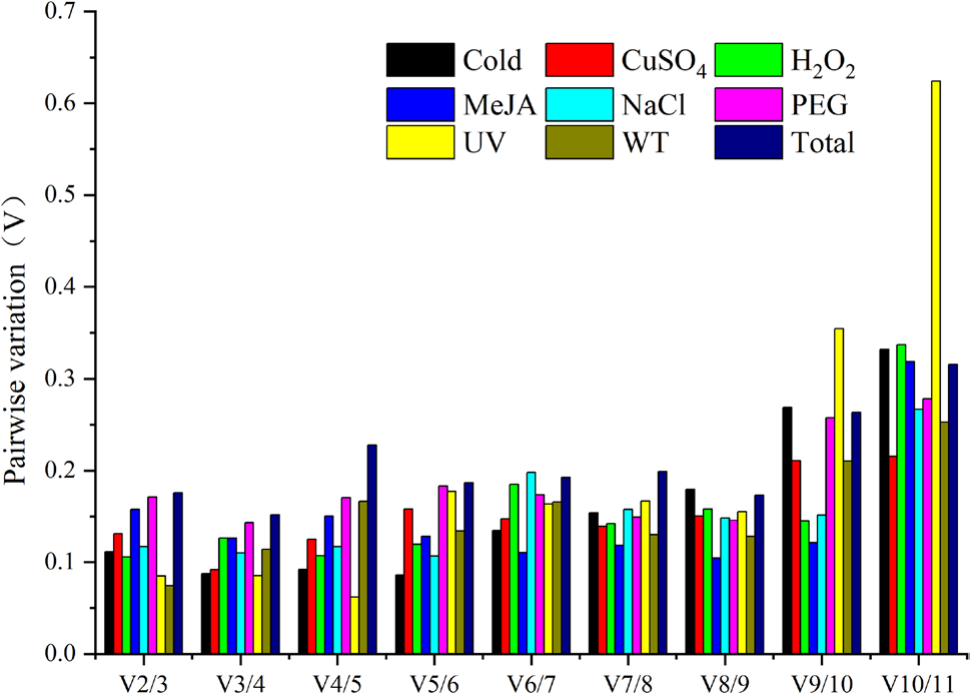
Determination of the optimal numbers of reference genes for normalization by pairwise variation (V_n/n+1_). Different treatments are marked as square frame with different colors. The cut-off value is 0.15 and used to determine the optimal number of candidate reference genes for qRT-PCR normalization.

geNorm analysis: The geNorm analysis evaluated the stability of all the 11 candidate reference genes using the M value (reference expression stability measure). These M values were calculated from the average variation of the gene relative against other candidate reference genes, and the lower M values indicated a higher gene expression stability^21^. As illustrated in Fig. 2, all the candidate reference genes had different levels of stability under different treatments. Here, the M value of *PP2A* and *SAND* was the lowest in most treatments and was deliberated as the most stable reference genes. Besides, *ACT* showed good stability under H_2_O_2_, MeJA, NaCl, and UV treatments, whereas *CYP2* was one of the most stable reference genes in the CuSO_4_ and UV treatment groups. On the other hand, the stability of *NCBP2* seemed to be less satisfactory than other candidate reference genes. Subsequently, the geNorm algorithm can also determine the optimal number of normalized reference genes^21^ by calculating pairwise mutations (V_n/n+1_). Normally, the ideal paired variation (V) score is less than 0.15, which means that the addition of any other gene will not have a substantial impact on standardization. In our study, in the NaCl, Cold, UV, H_2_O_2_, CuSO_4_, and WT subsets, their pairwise variation values (V _2/3_) were all less than 0.15. This showed that the addition of a third reference gene lacked significant effects on the normalization of the target gene, thus, the number of the optimal reference genes that were determined was two. In contrast, as shown in Fig. 3 and Table S3, the pairwise variation values (V _3/4_) of the PEG and MeJA subsets, was also less than 0.15. This indicated that the number of reference genes that were most suitable for these two treatments was three.

NormFinder analysis: To normalize raw data and measure the expression stability of candidate reference genes through intra- and inter-group variation, the NormFinder analysis uses the 2^−ΔCt^ method. Like the geNorm analysis, its lower values show higher stability. Table 2 shows the ranking of all candidate genes as calculated using the NormFinder algorithm. Among the PEG, Cold, and 'total' treatment subsets, it was observed that *SAND* was the most stable candidate gene, and also ranked higher in other subsets. After MeJA treatment (0.078) on all sample subsets, *TUBA* was deliberated as the most stable candidate gene. Besides, among the other five groups (NaCl, UV, H_2_O_2_, CuSO_4_, and WT), *PTBP* (0.670), *TIP41* (0.307), *GAPDH* (0.495), *CYP2* (0.483), and *UBQ10* (0.451) were observed to be the most stable genes, respectively. Similar to the geNorm analysis, our study found that the *NCBP2* was the most unstable candidate gene.

**Table 2:** Stability rank of 11 candidate genes by NormFinder.

Rank	NaCl	PEG	Cold	MeJA	UV	H_2_O_2_	CuSO_4_	WT	Total
1	*PTBP*	*SAND*	*SAND*	*TUBA*	*TIP41*	*GAPDH*	*CYP2*	*UBQ10*	*SAND*
	(0.670)	(0.319)	(0.146)	(0.078)	(0.307)	(0.495)	(0.483)	(0.451)	(0.568)
2	*PP2A*	*PP2A*	*ACT*	*PTBP)*	*PTBP*	*PTBP*	*SAND*	*SAND*	*PP2A*
	(0.672)	(0.399)	(0.313)	(0.452	(0.606)	(0.528)	(0.500)	(0.656)	(0.624)
3	*ACT*	*UBQ10*	*TUBA*	*SAND*	*CYP2*	*TIP41*	*UBQ10*	*GAPDH*	*PTBP*
	(0.676)	(0.560)	(0.407)	(0.472)	(0.615)	(0.594)	(0.535)	(0.672)	(0.696)
4	*SAND*	*ACT*	*PP2A*	*GAPDH*	*PP2A*	*PP2A*	*GAPDH*	*TIP41*	*ACT*
	(0.714)	(0.616)	(0.418)	(0.502)	(0.744)	(0.706)	(0.571)	(0.692)	(0.772)
5	*TIP41*	*EXP-1*	*PTBP*	*UBQ10*	*SAND*	*SAND*	*ACT*	*PTBP*	*GAPDH*
	(0.719)	(0.739)	(0.493)	(0.502)	(0.776)	(0.766)	(0.597)	(0.699)	(0.918)
6	*UBQ10*	*PTBP*	*CYP2*	*PP2A*	*ACT*	*CYP2*	*PP2A*	*PP2A*	*UBQ10*
	(0.730)	(0.869)	(0.667)	(0.641)	(0.851)	(0.855)	(0.604)	(0.728)	(1.198)
7	*GAPDH*	*GAPDH*	*GAPDH*	*ACT*	*GAPDH*	*UBQ10*	*TIP41*	*CYP2*	*CYP2*
	(0.999)	(0.957)	(0.998)	(0.675)	(0.954)	(0.960)	(0.773)	(0.815)	(1.275)
8	*CYP2*	*TIP41*	*UBQ10*	*TIP41*	*UBQ10*	*ACT*	*PTBP*	*ACT*	*EXP-1*
	(1.014)	(1.086)	(1.032)	(0.699)	(1.176)	(1.044)	(1.075)	(1.011)	(1.373)
9	*EXP-1*	*TUBA*	*EXP-1*	*CYP2*	*EXP-1*	*TUBA*	*EXP-1*	*TUBA*	*TIP41*
	(1.075)	(1.398)	(1.505)	(1.085)	(1.210)	(1.284)	(1.222)	(1.089)	(1.422)
10	*TUBA*	*CYP2*	*NCBP2*	*EXP-1*	*NCBP2*	*EXP-1*	*TUBA*	*EXP-1*	*TUBA*
	(1.528)	(2.677)	(2.711)	(1.324)	(3.510)	(1.291)	(2.200)	(2.013)	(2.635)
11	*NCBP2*	*NCBP2*	*TIP41*	*NCBP2*	*TUBA*	*NCBP2*	*NCBP2*	*NCBP2*	*NCBP2*
	(2.918)	(3.037)	(3.636)	(3.498)	(6.852)	(3.698)	(2.350)	(2.764)	(3.449)

BestKeeper analysis: Here, the raw data of the Ct value was directly calculated and the more stable candidate reference genes was evaluated by calculating the standard deviation (SD) and the Ct value^22^. Notably, lower SD and Ct values indicated higher gene expression stability, particularly when the SD was greater than 1 which indicated that the reference gene was unstable^23^ and could not be utilized for normalization. Table 3 shows that in the NaCl, MeJA, UV, WT, and 'total' treatment subsets, the *PTBP* gene was considered the most stable. However, the MeJA and 'total' treatment subsets were postulated to be expression-insensitive since their SD values were exceed 1 and could not be used for normalization. For this reason, such values should be excluded. Besides, under H_2_O_2_, CuSO_4_, Cold, and PEG treatments, the most stable genes were observed to be *CYP2*, *SAND*, *TUBA*, and *PP2A*. Generally, as illustrated in Table 3, it was observed that the *NCBP2* gene was still the most unstable under all treatments.

**Table 3:** Rank of 11 candidate genes by BestKeeper.

Rank	NaCl	PEG	Cold	MeJA	UV	H_2_O_2_	CuSO_4_	WT	Total
1	*PTBP*	*PP2A*	*TUBA*	*PTBP*	*PTBP*	*CYP2*	*SAND*	*PTBP*	*PTBP*
	0.97 ± 0.29	2.73 ± 0.65	2.50 ± 0.68	4.22 ± 1.23	0.54 ± 0.15	3.45 ± 1.07	1.04 ± 0.26	1.10 ± 0.30	4.63 ± 1.32
2	*PP2A*	*PTBP*	*CYP2*	*SAND*	*SAND*	*SAND*	*CYP2*	*SAND*	*SAND*
	1.76 ± 0.46	2.89 ± 0.82	2.76 ± 0.80	4.92 ± 1.30	0.87 ± 0.22	3.70 ± 1.03	1.13 ± 0.31	1.17 ± 0.28	5.40 ± 1.40
3	*SAND*	*SAND*	*PTBP*	*TUBA*	*PP2A*	*TIP41*	*UBQ10*	*ACT*	*EXP-1*
	2.23 ± 0.63	3.17 ± 0.81	3.44 ± 0.94	6.08 ± 1.70	0.89 ± 0.20	3.98 ± 1.13	1.83 ± 0.42	1.19 ± 0.23	6.33 ± 1.70
4	*ACT*	*EXP-1*	*GAPDH*	*TIP41*	*CYP2*	*PP2A*	*PP2A*	*PP2A*	*PP2A*
	2.32 ± 0.56	3.88 ± 1.04	3.92 ± 0.98	6.30 ± 1.69	1.21 ± 0.33	4.27 ± 1.11	2.03 ± 0.47	1.34 ± 0.30	6.34 ± 1.52
5	*CYP2*	*UBQ10*	*ACT*	*UBQ10*	*ACT*	*PTBP*	*ACT*	*CYP2*	*CYP2*
	2.48 ± 0.77	4.52 ± 1.05	4.07 ± 0.86	6.35 ± 1.65	1.49 ± 0.32	4.27 ± 1.30	2.51 ± 0.51	2.73 ± 0.73	6.35 ± 1.84
6	*UBQ10*	*GAPDH*	*SAND*	*GAPDH*	*TIP41*	*GAPDH*	*PTBP*	*UBQ10*	*GAPDH*
	2.91 ± 0.74	4.52 ± 1.19	4.31 ± 1.08	6.39 ± 1.71	3.90 ± 0.98	4.44 ± 1.29	2.54 ± 0.71	3.20 ± 0.68	6.88 ± 1.82
7	*EXP-1*	*ACT*	*PP2A*	*CYP2*	*UBQ10*	*ACT*	*TIP41*	*TUBA*	*TIP41*
	3.16 ± 0.88	4.81 ± 1.05	5.04 ± 1.17	6.46 ± 1.94	3.94 ± 0.87	4.87 ± 1.18	3.09 ± 0.74	3.63 ± 0.92	7.27 ± 1.90
8	*TIP41*	*TIP41*	*EXP-1*	*PP2A*	*GAPDH*	*UBQ10*	*GAPDH*	*TIP41*	*ACT*
	3.96 ± 1.12	5.19 ± 1.35	5.15 ± 1.39	6.80 ± 1.70	4.52 ± 1.15	5.05 ± 1.34	3.30 ± 0.83	4.05 ± 0.99	7.30 ± 1.59
9	*TUBA*	*TUBA*	*UBQ10*	*ACT*	*EXP-1*	*TUBA*	*EXP-1*	*GAPDH*	*UBQ10*
	4.32 ± 1.28	5.87 ± 1.64	5.91 ± 1.47	7.24 ± 1.62	5.09 ± 1.36	6.37 ± 1.93	4.10 ± 1.03	4.15 ± 1.01	7.30 ± 1.76
10	*GAPDH*	*CYP2*	*TIP41*	*EXP-1*	*TUBA*	*EXP-1*	*TUBA*	*EXP-1*	*TUBA*
	4.36 ± 1.27	6.49 ± 1.93	9.03 ± 2.34	7.96 ± 2.20	15.44 ± 3.98	6.71 ± 1.93	5.34 ± 1.44	7.75 ± 1.94	7.30 ± 2.02
11	*NCBP2*	*NCBP2*	*NCBP2*	*NCBP2*	*NCBP2*	*NCBP2*	*NCBP2*	*NCBP2*	*NCBP2*
	23.24 ± 2.56	21.11 ± 2.17	17.41 ± 1.52	21.69 ± 2.06	27.51 ± 3.12	24.04 ± 4.27	19.57 ± 1.98	22.34 ± 2.55	8.19 ± 0.82

Delta Ct analysis: This method evaluated gene expression stability by calculating the mean standard deviation (SD) of each gene. Here, the smaller the value, the higher the stability^24^. As shown in Table S4, the results of this analysis are consistent with the geNorm analysis. The only difference is that in the Cold and H_2_O_2_ subsets, *TUBA* and *PP2A* candidate genes are the most stable respectively. According to the geNorm analysis, the two most stable candidate genes are *SAND* and *ACT*. Hence, *SAND* and *PP2A* are the most qualified reference genes.

**Figure 4:**
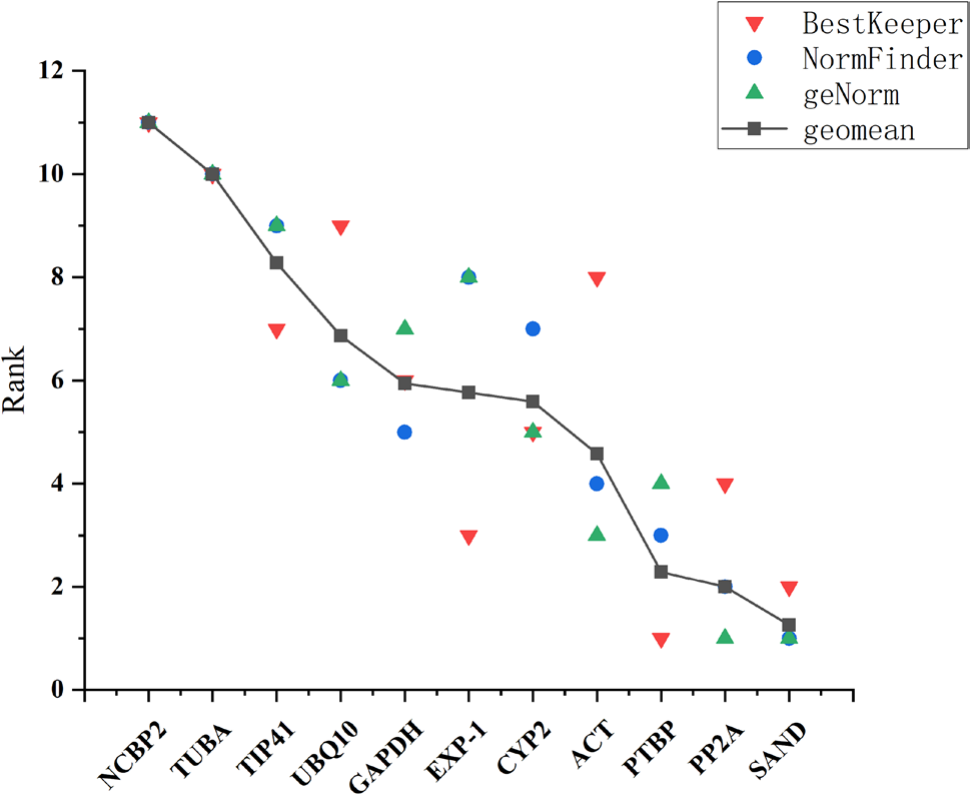
Comprehensive ranking of BestKeeper, NormFinder, and geNorm. Using BestKeeper, NormFinder, and geNorm algorithms, the 11 candidate reference genes were ranked comprehensively, and the geometric mean of each reference gene was calculated.

RefFinder analysis: As shown in Fig. 4, we further calculated the geometric mean of the ranking of each candidate gene using the RefFinder algorithm (http://150.216.56.64/referencegene.php#). This was based on the results obtained from the three statistical algorithms such as geNorm, NormFinder, and BestKeeper. Tables S5 and S6, show the comprehensive index ranking, whereby, the smaller the index, the more stable the gene expression^19^. This study showed that *SAND* and *PP2A* ranked the highest in most subsets, whereas *NCBP2* and *TUBA* ranked the lowest, making them the most unstable reference genes. In contrast, in the MeJA and Cold subsets, *TUBA* seemed to be a relatively stable reference gene. Despite the different assessment methods, this resulting difference is reasonable and acceptable. In summary, the stability of these 11 candidate reference genes from the highest to the lowest is: *SAND*, *PP2A*, *PTBP*, *ACT*, *CYP2*, *EXP-1*, *GAPDH*, *UBQ10*, *TIP41*, *TUBA*, and *NCBP2*. These results were similar to those obtained from the geNorm and NormFinder analysis, but slightly different from those of the BestKeeper analysis.

## Discussion

Currently, a majority of medicinal plants enhance the bioactivity of their medicinal components by inducing and regulating functional genes present in their biosynthetic pathway^25^. Several reports have shown that in plants, there is a significant correlation between the synthesis and accumulation of secondary metabolites and the expression levels found in the functional pathways of their biosynthetic pathways^26,27^. Coumarins are one of the main active components extracted from *A. decursiva*, which is one of the main sources of coumarin found in China and is listed as a special coumarin resource by the Chinese Pharmacopoeia. However, there exist no previous reports on RT-qPCR studies, which confines the research on the biosynthetic pathway of coumarin compounds from *A. decursiva*.

Here, we screened a total of 11 potential reference genes from the transcriptome data of *A. decursiva* (unpublished). First, as shown in Table 1 and S1, their primer specificity and PCR experimental conditions were confirmed, whereby the single peak of the dissolution curve exposed the specificity of these 11 pairs of primers (Fig. S1). As illustrated in Fig. 1 and Table S2, the average Ct-values for the 11 candidate reference genes is between 8.04 and 32.65, and the majority of these values were found to be between 23 and 27, which means that most of the 11 candidate reference genes were likely to afford accurate normalization^20^. The *SAND* and *PTBP* genes have the lowest variability, which suggests that they could have stable expression levels under different treatments. Conversely, considering the complexity of the ambient environment, the expression stability of these reference genes under different conditions must be investigated further^28^. Therefore, there is a need to use more statistical tools and conduct further analyses.

To ensure the accuracy and reliability of experimental data, four commonly used statistical algorithms (geNorm, NormFinder, BestKeeper, and Delta Ct) were combined for reference gene selection. Subsequently, RefFinder analysis was used to calculate the geometric ranking mean of each candidate reference gene, and then a comprehensive index ranking was done. Here, the results from this analysis showed that the ranking results acquired using different statistical algorithms were not similar, and the candidate reference genes also showed different levels of stability under different treatments. For example, Table 2 shows that *TUBA* is the best reference gene in the MeJA subset, and this is consistent with the results obtained when *Atropa belladonna*^29^ was exposed to different hormone treatments which showed that it had the most stable expression. On the other hand, when this gene was exposed to other treatments, its stability was unsatisfactory. This is most likely because some genes belonging to the tubulin family and that express isomers in specific developmental stages or tissues are regulated by different developmental processes^30^. Similar to the results from tomato^31^ and *Arabidopsis thaliana*^32^, *UBQ10* is the most stable gene in the WT group (Table 2). Also, the *PTBP* gene performed well in the UV subset, unlike in the Cold, PEG, and CuSO_4_ which is illustrated on Table 2 and S5). This result contrasted with the most stable performance of the *PTBP* gene in Cold, PEG, and CuSO_4_ subsets in *Peucedanum praeruptorum*^20^. Table 3 shows that under cold stress (4 °C) and the 'total' group, the *TUBA* and *PTBP* genes were the most stable reference genes, respectively, but this was different from the results obtained from geNorm and NormFinder analyses which showed that *SAND* was the gene with the best stability. Since various calculation principles are inconsistent, even under the same conditions, the ranking of these genes could be different. Therefore, the single software analysis has certain disadvantages when evaluating the stability of the reference gene. Our study recommends that a comprehensive evaluation and analysis need to be used to ensure the reliability and accuracy of the results. Besides, it is necessary to flexibly select the most appropriate reference gene for different experimental treatments.

Also, bearing in mind that a single reference gene could cause errors in the expression level of the target gene^33,34^, the geNorm statistical algorithm calculates the pairwise variation (V_n/n+1_) to attain the optimal number of reference genes^35^. Notably, the ideal pairwise variation value must be lower than the critical value of 0.15^33,36^. For instance, as illustrated in Fig. 3 and Table S3, the pairwise variation V _2/3_ in the Cold, NaCl, CuSO_4_, H_2_O_2_, UV, WT, and the 'total' treatment subsets, is less than 0.15. This demonstrated that only two genes are required to complete the gene expression normalization under these conditions. Consequently, it was observed that the *PP2A* and *ACT* are relatively stable reference genes under the treatment of MeJA, their pairing value V _3/4_ was < 0.15, and their optimal number of reference genes was three.

Taken together, the analysis results from the three algorithms (geNorm, NormFinder, and Delta Ct) seemed to be consistent, whereas those of BestKeeper are different. Figure 4 and Table S5, confirm that under most external conditions, the stability of *SAND* and *PP2A* genes are significantly better than that of other reference genes, whereas *NCBP2* and *TUBA* are nearly always defined as the most unstable reference genes. Of note, the *SAND* and *PP2A* are new reference genes that were identified through screening the data from the Arabidopsis gene chip. To date, many studies choose the *SAND* and *PP2A* genes for their reference gene standardization studies. For instance, studies that used *Arabidopsis thaliana*^32^, tomato^37^, *Petunia hyrbrida*^38^, *Peucedanum praeruptorum*^20^, etc. Conventionally, reference genes, such as *TUBA*, *GAPDH*, *UBQ10*, and *ACT* play a housekeeping role in the maintenance of cell structure or primary metabolic activities. Nevertheless, increasing research indicates that the expression levels in most of these traditional reference genes can somehow vary greatly and in many species, they are unsuitable for gene normalization under specific conditions^39,40^. Therefore, given a subset of experimental conditions, the housekeeping genes used as reference genes in each different species should be handled carefully^41,42^.

## Materials and methods

### Samples preparation and treatments

Here, one-year-old plants of *A. decursiva* were collected from Ningguo City, Anhui Province, China (longitude: 118.95E, latitude: 30.62 N), and was identified as *Angelica decursiva* (Miq.) Franch. et Sav. by Zhang Ning, school of pharmacy, Jiangsu Health Vocational College, and deposited in the herbarium of the medicinal botanical garden (ID: JSJK-AD-021). The planting field of *A. decursiv*a was a private land and the landowner has allowed us to collect plant materials for further study. The collection of plant material is in full compliance with relevant institutions, national and international guidelines and legislation. Then, these accessions were transplanted into plastic pots that contained a mixture of vermiculite, perlite, and peat moss (1:1:1 v/v). Next, the plants were grown in a greenhouse at a temperature of 25 °C, a long photoperiod of 16 h light and 8 h darkness, 40–65% relative humidity, and 3000 lx light intensity until treated. For drought treatments, a 200 mL solution of 25% PEG 6000 (w / v, polyethylene glycol, Sangon, China) was used to treat the plants for one week. For salt stress treatment, an approximate amount of 200 mL (600 mM) of NaCl was used to water the plants for 7 days. For cold stimulation treatments, the plants were incubated at 4 °C for 48 h. To study hormone therapy, 25 mM MeJA was applied for 6 h. To assess heavy metal stress, 500 mM of Copper sulfate (CuSO_4_) was applied to the plants for 24 h. In the case of oxidative stress, 50 mM H_2_O_2_ was used for 24 h. To induce ultraviolet (UV) light, these plants were irrigated using distilled water (100 mL) and then exposed to ultraviolet light for 24 h. Notably, all the above treatments had three biological replicates. The remaining plants that received no treatment served as controls. Lastly, all samples from each treatment were washed with MINIQ- filtered water, quickly frozen in liquid nitrogen, and stored at − 80 °C.

### Total RNA isolation and cDNA synthesis

About 100 mg of the different frozen tissue samples were used to extract total RNA using the Spectrum Plant Total RNA kit (Sigma, USA). Next, the quality and purity of the extracted total RNA was determined using the NanoDrop spectrophotometer 2000 (Thermo Scientific, USA), and its integrity conf*irmed on a* 1.5% agarose gel. Here, only RNA with A260/280 ratios between 1.8 and 2.2 and an A260/230 above 2.0 were used for cDNA synthesis. Subsequently, RNA samples were pre-treated with RNase-free DNase I (Takara Biotechnology, Dalian, China) to remove contaminating traces of genomic DNA and then used for reverse transcription. Lastly, following the instructions from the HiScript Q RT SuperMix for qPCR (Vazyme, China) was used for first-strand cDNA synthesis with oligo (dT) as the primer.

### Primer design and RT-qPCR conditions

Here, a total of 11 candidate reference genes were selected from the transcriptome data of *A. decursiva* (unpublished). Next, the built-in program in the Bioedit Sequence Alignment Editor (v7.0.9) was used to screen and select potential single genes using local blast (TBLASTN). Subsequently, the corresponding homologs of these reference genes were selected from the database of The Arabidopsis Information Resource (TAIR) (http://www.arabidopsis.org), and only single genes with lower E-values and higher scores were selected for subsequent analysis. Table S1, shows the reference gene IDs, homologous loci, gene sequences, and the different expression levels (FPKM, Fragments Per Kilobase per Million) of all the 11 candidate reference genes.

To avoid amplification of any contaminating genomic DNA, primers were designed to cross at least one intron/exon border that contained both donor and acceptor sites, and then exon analysis was performed using the AlignX program in the vector NTI advance 11.5 package. Subsequently, the Primer3Plus (http://primer3plus.com/cgi-bin/dev/primer3plus.cgi) was used to design primers with the following characteristics: amplicon length was 100 to 150 bp, GC content was between 40 and 60%, primer length was 18 to 24 bp, temperature difference of each primer pair was less than 1 °C, and the melting temperature (Tm) was between 55 and 60 °C. Consequently, all the primer pairs were tested using conventional PCR to determine the combination of forward and reverse primers that performed optimally and the resulting products were examined using 1.0% agarose gel electrophoresis. Besides, from a series of standard curves of five different cDNA dilutions, the amplification efficiency (E) and correlation coefficient (R^2^) were calculated. Table 1 lists all the gene-specific primer pairs that were designed and used in the RT-qPCR analysis.

### Stability evaluation of candidate reference genes

To obtain the RT-qPCR data, three biological and technical replicates were done for each sample, and all data presented as mean ± standard error of the mean (SEM). Consequently, statistical analyses were performed using the Student’s *t-test*. Next, representative graphs were generated using OriginPro 9.1 (OriginLab Corporation, Northampton, MA, USA). Subsequently, data analysis was performed using GeNorm (ver.3.5), NormFinder (ver.0.953), BestKeeper (ver.1.0), and the Delta Ct method following the instructions from the manufacturer. Lastly, a comprehensive stability ranking analysis was performed using RefFinder (http://150.216.56.64/referencegene.php).

## Conclusions

Selecting appropriate reference genes is a significant prerequisite for quantifying gene expression using RT-qPCR. This is a conducive research on the key enzyme genes that are involved in the biosynthesis of coumarins and other interesting secondary metabolites found in *A. decursiva*. So far, this is the first systematic screening experiment of the most suitable reference genes for *A. decursiva* under various external treatments, like cold stress (4 °C), drought stress (20% PEG), Methyl jasmonate (MeJA), salt stress (0.5 M NaCl), oxidative stress (H_2_O_2_), ultraviolet (UV) induction, metal stress (CuSO_4_), untreated (WT), and 'total' (all treated samples). Our results have exposed that the 11 candidate genes have different stability in *A. decursiva* when exposed to different experimental treatments. From the overall stability ranking, *SAND* is the most stable candidate reference gene, followed by *PP2A* and then *PTBP*. On the other hand, the *NCBP2* gene has the lowest stability making it unsuitable for further research. In summary, the reference genes evaluated in this study can be helpful for accurate normalization of the RT-qPCR data and any other future work on the gene expression of coumarin synthesis present in *A. decursiva*.

## Supplementary Information

Supplementary Information.

## Data Availability

The datasets generated and/or analyzed during the current study are available from the corresponding author on reasonable request.
