# Supplementary material for: Evaluation of Angelica decursiva reference genes under various stimuli for RT-qPCR data normalization
Source: Sci Rep. 2021 Sep 23;11:18993. doi: 10.1038/s41598-021-98434-6 (PMC8460625; doi:10.1038/s41598-021-98434-6)
Supplement: Supplementary file 1 — Supplementary Information. [file 41598_2021_98434_MOESM1_ESM.docx]

Research article

Evaluation of *Angelica decursiva* reference genes under various stimuli for RT-qPCR data normalization

Yuedong He, Yuan Zhong, Zhenzhen Bao, Weiqi Wang, Xiaoqing Xu, Yanan Gai & Jie Wu

Supplementary figures and tables


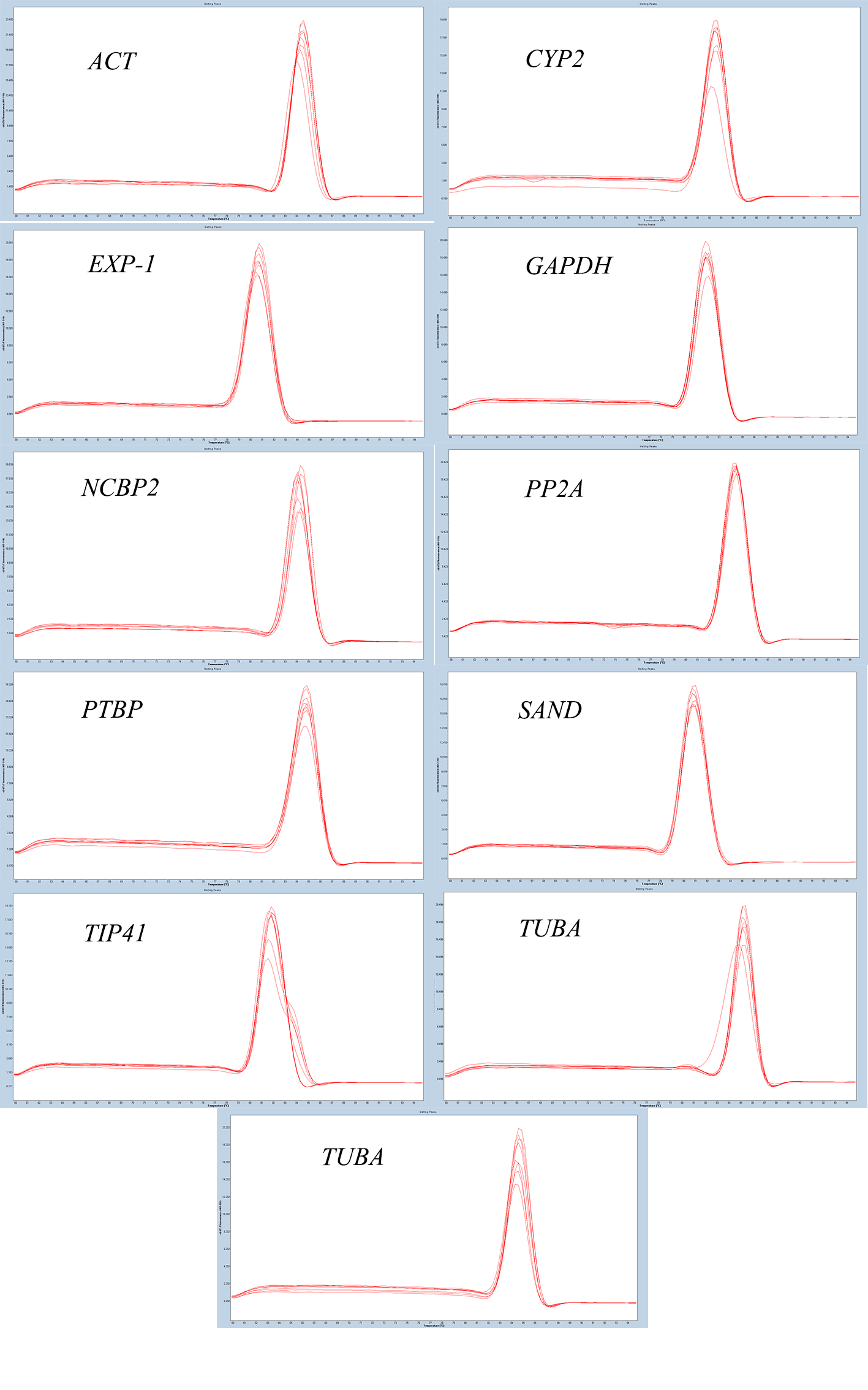
Figure S1. Melting curves of 11 candidate reference genes tested in this study.

Table S1. Informations on 11 candidate genes of *A. decursiva*.

**Gene abbreviation**-*CYP2*. Gene ID-Unigene27688. **Homolog locus**-AT4G33060. **Length**-1655. **Gene sequence**-TGCATATGTCCAAATTTGACCATGGTTAGAAACTAGTCACTCTTTAGTTAAAAAACACACACACAAAACACTTACAACTCTCTCCGATCTCTCCACCGTCGTTGATCTCTCTCCGGCTTGAAGGTGGGAGCTAGACGGGAACTCAGGCTCGTGCCTTGTGATGTCAGGTGTAGCTTGACAAGTGCACGTTTGTATGCTGTGTTCAGTTATAGAAAATATGGGTTCTTTCTGCAGTAAACAGCGACATAATCAAGCTGATTCCGAAGAAAATGCACAGACTGCAGAAATAGAAAGGCGAATTGAGCTAGAAACAAAGGCAGAGAAACATATACAGAAGCTTCTTTTACTTGGTGCTGGAGAGTCGGGAAAATCAACAATCTTTAAGCAGATAAAACTTCTATTTCAGACCGGCTTTAATGAGGCTGAGCTAAAGAGCTACATTACAGTTATTCATGCAAATGTGTATCAGATAATAAAAATATTATATGATGGGGCAAAAGAATTGGCTCAAAATGAAGAAGAATCCTCAAAATATGCTTTATCGGATGAAATCAAGGAAATAGGAGAGAAACTATCGGAAATTGGTGGCAGGTTGGATTATCCTTGCCTCAGTAAAGAGCTTGCTCAAGAAATAGAAACTCTCTGGAAAGACGATTCCATACAGGAAACATATTCTCGGGGTAATGAACTCCAAGTTCCTGACTGCACTCTTTATTTCATGGAAAATTTACAAAGGTTGGCTGTTGCGGATTATATTCCGACAAAGGAGGATGTTCTTTATGCAAGGATCCGTACAACTGGTGTTGTAGAAATTCAGTTCAGCCCGGTTGGAGAGAACAAAAAAAGTGGCGAAGTATATAGATTATTTGATGTTGGAGGTCAGAGAAATGAGAGAAGGAAATGGATTCATCTGTTTGAAGGTGTTACAGCTGTGATTTTTTGTGCTGCCGTTAGTGAGTATGATCAGACTCTTTTTGAGGACGAGAACAGGAACAGAATGATGGAGACAAGAGAACTCTTTGAGTGGGTCCTCAAGCAGCCATGTTTCGAGAAAACATCCTTCATGCTATTTCTGAACAAATTTGATATATTTGAGAAGAAGGTTCTAGATGTGCCACTAAATGTATGCGATTGGTTTAAAGATTACCAACCAGTATACTCGGGAAAGCAGGAAGTCGAGCATGCATATGAGTTTGTCAAAAAGAAATTTGAGGAGCTATATTTTCAAAGCACTGCCCCTCACTGTGTGGACCGAGTTTTTAAGATATATAGGACAACTGCTCTTGATCAGAAGCTTGTGAAAAAGACTTTCAAGCTGGTAGACGAGACCTTAAGAAGGCGAAACCTCTTCGAAGCGGGTTTGTTGTGATAGTGGATTTCATCCGAAAGGAAATGTTAATATCTTGAAAAGCTAGATTCCTGCAGTGAGGTGGGAACACTCATTAAACTCTTTACACCGTAATTTACAGGTAAAAAATTGGCTGTTTGGTATACCCTCTGTAATAGGAACTTGATGTTACCCCTGTTTCTTTTTTCCTTAAAAGAAGTCCCCATGATTTTGTTTTCTGTTTGTAGCATACCGCTAGTTGGAGAAGCAAGCAAGTATATCAAAAGTTTTTTTATACCTTAATTTCGTTTTTGTTTTTTTACAGTGA

**Gene abbreviation**-*EXP-1*. Gene ID-CL3447.Contig1. **Homolog locus**-AT2G32170. **Length**-1337. **Gene sequence**-GTCAAAATAATCCTCTCTTTTTGGAGACCCAAGTAAGAGCCTAAACTTTTCCCCGACTAGAATTAGTATCCCTAATATATTCTAAATTAAAATTCAGAAAGACAAAGAAAGTCAAAACTTATACTTTCTGTTCTGCTTTATTCCATTATTTGTGCAAATAAATTATTTAGAGTTCTAATCTATAGGCTTGACACTATATAATGGTGATCAACTGACCCAAAACTGTGATGATCCATGTATTGCACTCCCTTATCAAGGACATTCAAGCTGATGACCTGGCCCAGAAGTCTCGGGTAAGATGTTCCAACCGAATAGTAATGAAGTGCAGCAATATTTAGCCTCAATCAAGGCAGCAAGTTCCCTGCTGCTCCCTTCCGCATCGTCCAAAATGCAGTGTAGTAATGGTTTTGCATCATTGATCGAGGATTTGTGGTATATGTGGTCTCAATTGTCTTTTCATTCTCTAACTGAAATCCATAATGAAAAGCAACTTTTTTCACGTCCTCCAAACTAAGTTCGACTGACATTTCGTCTTCTTGACTATCTGCAAAGTGATAAAGCAAGGGGCCCAAGTTTATCCACACACCACCGTCCTTTAGAATTCTAGAAATTACTTCAATATATTCAACAATGTTGTGCCCTGTATCAAGGAAGAAACAGGTTACAACTGCATCCCAAGCTCCTACTTGGCTTGGATCACAGTAAACTTTAACAAAATCACCACCACGCATGGAAAAGCCTTCAGTTTTTCCTGCGCTGGCAGGATGAATCTCCGGTATTGAAATAGGACGAAGCTGGTCACTGTCAGAAAGTGAATTGCAATTGCTATGAATCCAAGGATGGATAGTCCATTCATTAGCCCTTTCCGCTTGGTTAAGAATAAAACTCGAGCAGATCATCATATAGTATGAAAATTCATTTCCTTGGCTTGCAAAACCAAGACATGAAATTTCCAAAGCCAGTCTACCAAGTCCAGCACCCGGAACTAAACATGCAGGAGGGCTCCCTTTACTTCGATTAGGAAATTGTGATTTAAGCTCTTCAAGAATAGGCTTATAGCACTGATCACGTTCTTGTTGTCCCTCTGCAGCCCAATCTCTTACAATATTTCTTATAATACATCGAACCTTATCTACATCAACTAGAGGAACATGCGACTGCAGTGATGGATCCAACCAATCAGGAGATGAAGACACATTATAATTGGAGTCTGCATTAGAGTACGAAGTTTCCTGAATCCTAACACCATTGTTACCAGCCTTATCTGCTTTGGTATCACATTCCAAAACATGACGATTCCCAGTGTCAGACTCAATGAAAAGCCCAAGTTTCTCCTC

**Gene abbreviation**-*GAPDH*. Gene ID-Unigene21826. **Homolog locus**-AT1G42970. **Length**-1651. **Gene sequence**-TAATGAGGAGTTCAATTAACCTGCATGATAGAGCTGAAGTAAAATATAAGTGTATTATGTATCAACACTGAAAAATAAGAAGAAAAAAGTAGGTATTCTCTGAGTCTTTAGTTGTGCAGCAATGGCTTGTAAAGCAGCTCTGGTTTCCTCAAGCCTTCCAATAAATATTAGACTTCCATCTAGAAGCTCTTATTCTTTCCCTGCTCATTGCTCCTCCAAGAGACTTGAAGTAGCTGAGTTTTCCGGGCTCCGATCCAGTGTTTCGCTCACATACTCAAAGAATGCTAGTGAAGGATCTTTCTTTGATGTGGTGGCTTCCCAACTTTCTCCTAAGACTTCAGGATTATCCCCTGTTAGAGGAGAAACAGTTGCCAAATTGAAGGTAGCAATCAATGGTTTCGGGCGCATTGGCAGGAATTTTCTTCGTTGCTGGCATGGACGCAAGGACTCTCCACTCGAAGTTATTGTTGTCAATGACAGTGGTGGTGTCAAGAATGCATCGCACTTGCTGAAGTACGATTCAATGCTGGGCACTTTCAAGGCAGATGTTAAGATTGTAGACAGTGAGACCATCAGTGTTGATGGAAAGCACATCAAGGTTGTCTCCAACAGAGATCCACTCCAGCTTCCTTGGGCTGAGATGGGAATCGACATTGTTATCGAGGGTACTGGTGTGTTTGTTGATGGCCCTGGGGCTGGGAAGCACATCCAAGCAGGGGCAAGGAAAGTTATCATCACTGCACCAGCAAAAGGAGCCGATATTCCAACTTACGTTGTTGGCGTGAATGAGAAAGATTATGGTGATGACGTTGCTAACATCATAAGCAATGCTTCTTGCACTACAAACTGCTTAGCTCCTTTTGTGAAGATCTTAGATGAAGAATTTGGTATCGTTAAGGGAACCATGACAACCACTCATTCTTATACAGGAGACCAGAGGCTTTTAGATGCTTCTCATCGGGACTTGAGGAGAGCCAGAGCTGCAGCCTTGAACATAGTCCCAACAAGTACTGGTGCAGCCAAGGCTGTATCTTTAGTGCTACCCCAACTGAAGGGCAAGCTGAATGGCATTGCACTCCGGGTGCCCACGCCTAATGTATCCGTCGTTGACCTTGTTATAAATGTTGAGAAGAAAGGACTTGCTGCTGAAGACGTCAATGCTGCCTTTAGAAAGGCTGCTGAAGGACCACTGAAGGGAATTCTGGAGGTCTGTGATTTGCCTCTAGTGTCGGTGGATTTCCGGTGCTCTGATGTTTCATGCTCCATCGATTCATCATTGACAATGGTCATGGGAGATGACATGGTCAAGGTCGTAGCCTGGTACGATAACGAATGGGGATACAGCCAAAGAGTTGTAGATTTGGCGGATTTGGTAGCAAGCAAATGGCCAGGTGCAGCAGTAGCTGGAAGTGGTGATCCATTAGAAGATTTTTGCGAGACAAACCCTGCTGATGAGGAATGCAAAGTTTATGAATAATTTACCTTGTTCCACCTTTATTTTTCCAAATTAAGTTGTTTAGGTGGTTGTTTAGTTCAAGTCATTCGACGTGTTAGTGTCACTAATATGGAATAATCTACGGACTAGGGGACAGAAAATTTGATGATTTATTGTTGATGAGAATATTATGGGATTTGGAATATCGAGAAGAAG

**Gene abbreviation**-*NCBP2*. Gene ID-CL9038.Contig1. **Homolog locus**-AT5G44200. **Length**-1120. **Gene sequence**-GCAGTTTTAATTTGTCTCCTCTCTCCTCCGGCCTCCGTGTTTCGCCGCTCGGCACCAAGCTAGGTGATTTGCTCATTTTGTTGCTATACGTACCCTATGGCCTTACTGTTCAAGGATCCAAACAAGATCTCTGCGTACAGGGATAGAAGGTTTCAAGGTTCGCAAGAAGAATTTGAACATGCTCTCCTGACCTCAACAACTGTTTATGTTGGCAATATGTCCTTCTACACAACAGAAGAACAAGCATATGAACTGTTCTCTCGAGCAGGAGAAATCAAAAAAATAGTTATGGGATTAGATAAGAACACTAAAACTCCTTGCGGTTTTTGCTTTATCATGTACTATTCTAGAGAGGATACTGAAGATGCTGTTAAATACATAAGTGGGACAATACTTGATGATCGTCCTATTCGTGTAGATTTTGATTGGGGGTTTCAAGAAGGAAGACAATGGGGACGTGGTCGAAGTGGTGGCCAGGTACGCGATGAATATCGTACCGACTATGATCCTGGCAGGGGTGGTTATGGAAAATTAGTTCAGAAGGAGTTGGAAGCACAAAGGGAACTAGTAGATTACGGTGTAGGGTCTCTGGGCTCTTTTCCACCTGTCATGCCATCTAATTATGGTAGAAACAGTGGAAGACAGGGCTATGGTGGCTCCAGTCGACACGGTAGAGGAGATTATCAGCGGAAGCGGCATCGGGAAGATGACCGTTTCGGGCCAGAGATCACTAAAAGAACTTCAGATCATGATTCTCGTAGAAATTCTGATCATGACTCCAGACCGGAAAAGAATCCACGTTTCCGAGAGAGCGGTGATTCCGACGAGGAGGAAGATGATCGAAAAAGACGCCCTTAGCTGGTTACAAAATACTGACCTATGCTACATATCAATCCAGTGTTTGGATCCAGAAATTATTTATTTACATTTGCTTGTAACGTGGAGCATATAATATTGTATCTTAGGATAATATTTCATTGCACCGTTTCTCGGTTTTCTGATTTGCATCTTAGGACAACAGGGCATTGCAGTTGTTTCTCAAAATTTTATCGAATCGACAGATCTAGGGCATTGCAGTTGTTGCATGCATTGTGGTCCCCAACTATCTTTCATAACTCCG

**Gene abbreviation**-*PP2A*. Gene ID-CL7836.Contig4. **Homolog locus**-AT1G13320. **Length**-2098. **Gene sequence**-GTGCCTTGTTAGGCCGAAACTACCATCGAAGTTTCTTCCGCTGGTTTCGCAGAAAAAATTTGTTAATCTTACCAACAAAAAACATTCTCAGGCTACTAGTAGCCTGGTGTCTCCACATTTTGATAACAACATATTAGGGAGTACATTTAAAAGTTTAAAAGATTTCGAAACTAATCTGTGATAGCATCTTCAATCAGATTAGAACCTCTGATAGTATTTCCATATGTGAAATGAAACTCTAACTCGACATCATGGCATGACTAGCCTGGAGTGCTTCATTTGCGAAATAACGCACATCCACGTCTGGGTCCTCGGCAAGTTCCACAAGAGCAGGACGGATAGTGTTTTCAACCACGGATTGATCCACAATAGGGATAAGGGATTGTAACACCTTTGCTACATTGAACTTGATATTGGGCACTCTGTCCTTGGACAAAGTAACAATCACAGGCAGCAACTTAGAACAAGTGATTTCTGAGCCCATGACAGGAGCGAGTAGAGAAATTGCACGGAGGATAGTCATTCGATACAGGTAATGAGGGTTATTAATCATATCCAAAACCTGTGGAACAATATGCTGCATCGCCCATTCTGGACCAAATTCCTCTGCAAGGCGCTTCAAGTTATTGGCAGCGGCATCTCGAATTGAGTAAACCTTATCCTGCAACCACTGCATACAGAGAGTACCTAGTTTATCATCAAAAAATCCTACTCCAAGCTGACTAGCCAATAGGGGTATATACTCAATAATTGCCAGTCGCACTCTCCAATGTCGATCCTCTGCAAGCTCAACAATCGCTGGTAAGAGAGATTGGGATAATAAATCTATTCCAATAACCTGGTTGACTTGATCCAGCTTGCTGATAATATTGAGACGCACATCAGGAAATTCATCCTTTAGAAGAGAAAGAAATATTGGAAGAAGCTGTTCAATTGTTGCTTCCTTTCCTAATACAGGAGCCATGCCCATTATAACCGAGGCCAGAGCAGATCTAACATGCTGAGAAGAATCAGATGATAACTCCTTCACACATGGAAGGATGTGCTGAATTGCTAGCTCAGGATTAAGAATTCTGCAGAACTTGGTGACTTTGCCAGCAGCAGCTATACGTACTTCAGCTTCATTATCTCGAAGAAGTCGCACATATGCAGGAACTAATTCCGTCCTGGTAGGTTCAGGTCCCACAGCTTCACATAATTCATAGAGCTGATTGGCAACCATGTAACGAACACGCCACGACTTATCCTGGGAAAAGTTGACAATAACGGGAAGTATATGTGCTACACAATCTTGAGGTTCCAACAACTTTCCAAGAGCAGCACATCCTTCAACAGCTAGCAACCGAACAGAATCTTGATCATCCTGGGTAAGATCCTCAAATATTGACATGATATCAGTCTTGAGATGAGCAGGTTCAATGGTTGCAGCAAACTTCCCCAAATTTGTAGCAGCAGATCTCCTAACCATAGGCATGTCATCCTGACACAACTGACTGTATATCGATCGCAATTCGGTTTTCAACGTCTCTGGTGCACTGGGATATGCGATGTGAAACAATCCACAAGCAGAAACTCGAGCAGTAAACCATTCACCTGCTGCCAACCTCTTCACTAGAGGAACAAAATAGTCATTTAAATCACTCTCCCTCATCTGAGATCCAATTCTACAAAGCGACTCCACGGCTTTGTCCCTGACACAAGTTTCTTCAACAGTGCAAAGATTTTCAAGAGTGGGGAGCAAAACATGAGCGTGTTCCACACCTCCAACATAAGGAATAAATACTCCCAACTCTTCGGCCATTGCAAGAAGAACCTCATCGTCATCATCATTGTTCTCGCTCAAAAACGGAATCAATTCCCTTCTTGTCCGTTCCTCCCCGAGAGCACGAGCAATAGTCGAAAGCCTTCTAATAGAATTAAGTCGCAACTGAATATCGTCATTCTTCAGCTCATCAATCAACACAGCTATTGGGTACAATGGCTCCTCAACATTCGACATTGTATTTCACTAGATTCTTCAACGGTTTCAAGCTTTTTTGCAAAGAGGGAGGGAGGGAGATTAGTGCAGAGAGACGAGCGAGAGAGAGAGAGAGAGAGA

**Gene abbreviation**-*PTBP*. **Gene ID**- CL7808.Contig1.Contig4. **Homolog locus**- AT3G01150. **Length**-1713. **Gene sequence**-TATGCACACATATCCTTGGTTGGACACCAAAGGACAGTAAATAGAACAAAAACAATTTGAAGAATTCAAGTCTACTGAACAGCACTGATACAACTTACTATCTGTAGTAAGGTGGGTGGTGACTATGTGGGGAAGAACTATCATGTTGCATATTTGACTGATTCCCAGGCTGACCTCCACTTGCTGAAGAACTGGGATTGATTTGGCTGCTGTGTCCAAGAGGTGAGGAACCAGCAGGGGAGGCCATGGGATTATAAGCCTGACCAGAGGGAGGCCCATAAGTTTGTCCAGGTAATGTGTTAGGGCCTGAAACAAAGGAGGGTCTGCTAAGCTGCATGGCTGGATCCCAAGATGGAGTTTGTCCAGGCGGGCCCTGCATGGGAGGTCCGTGGCCAGGAGGTCCTTGCCCGGGAGGTCCGTGGCCAGGAGGCCCTTGACCGGGAGGGCCGTGGCCAGGAGGTCCTTGACCGGGAGGGCCGTGAGGCTGCCCATAATTGATACTAGAAAATTCGCTCCCAATAAAGACCGGAGCAGCATGAGGATTCTGCCATACTGTTGCTCCAGCAGGCAGACCAGCAGCAAAACCGGACTCTGGTACTGTATAATCCCTACTTTTATCGCTGAAGGCCTTTACATTGAGATCAGTATGACGAGAGTATGATATATGAAGCTTACAGTAGCCACCATCATATATGCAGTGTCCCTCTAGAGCATCTTTAGCTACAGCGGCAGTGTTGACATCAGGATACTGAATTAGAGCCTGAGTTGTCGCGTTCTTCTCGAATATTGCAATTTTCTGAACCGTGCCAAATGCAGAGAATACCGTGTTAAGAACATCTACAGTGACATCATAGATCCTATTTTCAATTGAGGCAAGAAGTACATTACTCTCCGGTTCTTTTTTCTTTCCGTCAGGACCTACAACAGGCTGGACAAATCCCTCAATTGCAGTTGCATTAACAGGAAGATATGGATTTGTATAGTCCCGGCTACGGTGTGATTGGAACTTGATGTTTAGATCTGTGTGAGCTGAATATGAGATGCGCAGATTGCAAGAACCAACATGTTCTGGAAGCAAGTACCTTGGAATACTTCTGCCATCTAAAGCTTCCCTTGCTGAAAGAGCAGTCTCAGCATCAGTAAACTGGATTAGTGCCTGAAAACCTGCTGCCTTCTCAAAAGTAGCAATCTTGTGCACAAATCCAAAAGCCGAGAAGACCAAGTGAATCACATCAATGCTTACATCACCGGCTTCTACACCCTCAATGGTTACCAGCAAGACATTTCCCGGAACATCACCTGGACCCTTGTTGTTGACAATTTCATGTCTGTTTGAATACTGTATATAAACATGTTTACCCCGAACAGATGCAGGTTCTGATGATGAAGCATAATATGAAACCATATTAATGGCCTGATTAAGATCCACAAATTCAACGAAGGCTTGATTGCGATTAGCGCCGACATTGCACTTGGTGTTAACGATCTTACCAAAAGGCTTGCAAAGCTCAACGAGCTCTTCTTCAATACACTCCCAAGGCAAGTTACGCAAGTGAAGCACTTTAGATGGAGTCTGTGTGTATCGAAATTGAGGCTGATTTGAATTCGACATCCGCTCACTTTTGATTCAATTAGGGTTTTGTTTGTGTGTGTACATATATTATTATTTGTATGGGTGGTGGTGAATATGCAGAGGAGAGCTGAACGTTTTTG

**Gene abbreviation**-*SAND*. **Gene ID**-CL11842.Contig1. **Homolog locus**-AT2G28390. **Length**-2403. **Gene sequence**-TGGTACATTAACAATTCAGGTGCTTCTATTGCAAAATTGTATGTTGGCCTCAATGAAGAGACTTAAATATACCTAGAGATGGATTACAGACTCAGCATACAAACATACAGTAAATGACAAATACATCTGATGATACATTCACGAACTAATAGGATTGATATATTACGATCGGCATAATCTTGCCGTGTTAGAACCTTATTCAAAAATCAAAACTAGTATACATGAATAACATAGTTTTCTGGAAACATCACCATGAAAAGGGGCTTGCTCCCAACAAAAAAACTTCATTTTCCACATCTTTAACCCATTGGCATACTCGGTTGCATGTCTTTATAGCCAGTGCCTTGTCTGCTAGAGGATCAAATGCTGCATAAAGTTCAAAATCCTGAGTAACCCAGCAGAGTAGAACATAGTTCTCGTCCCTTCTAAACTGGGTTTTGTGAGGACCAAGTTCTCTATCATGCATAGAAGTATACAGCTTCTGATATGCTCTATATAATCTTTTCTGTTGTTTAGGGGTGTTGATCGGTGATGAGAACTCAGAAGATACATATTGATCTAGATATATACTGCGGTAAACGAAGTGCCAAAGCCCAGCAGGACCACCTAACAGTGCCTTACACCCATCTGCAGAGTCCGGTGGGGGTCTAGGCTTACCTAAATGAGATGACAAAGATCCCGAGCGAGGAGATGGATTAACAGGCAAATCTTCGACACGCATGCCACCATCCAACATCGACCTTTGAGCTTCATTAAGAACATTTGACTTCAGAAGGACCATTTCGATACGGATCCTCCAATCTTTTAGACGATGAAATGCATCAGGATTAGCAGTAAGCAACATCAAATATGTATCAGCATCAAGATAATACACATAAGTATATAAAAATGCCATTGGATTGTATCTCGGAAGACAGATTGGAGAGAAAGATTCAGATGTCCTGAATGATTCAGATGACATCACAAAGTTGGCAAGCAAGAGCATATCATCGGGATGAAGAGATGCTTTTTGTGCACCAACCAGACTGATGACCTTGTGTTTACACATTAATATCGCGAAGAGGACACCTGACTCAGCAACATCCTGCAATATGGCACCGGCAGCTTGCCTTGTTGGATAAGCAAGGGGAAGACAAGAGTAGGCATGAAGAAAAGTGGCAGGGTTCCAACTAAACGAGTGGATGAGAGAAGAGAACACAGCATCTGTTCCCCCAAGCAAAGGTGTCATATCAAATTTCGGATTCTTCTCAAAGCATCTATTTATAGACTTTGTCAGAATAAGTATCATCTGGCCATAAAGAAGTTCCAGTTGTTCACTGAGGGATTCATGAGGCTCTTCTGTACAGCTTATGCAAACTAGATATATTGGTCCTTTAACAAGAAAAACCACCTGGTGTTTTCCCGCCCTAACCAACTTCACGCGATCTCCCCCATTCTCCACGAAGGAAATGATGGCTTGCAAAGTTGCTGAAAATCCTGCTAATCTGTGTTCATCTCCATATCTTGAATATATTGGTTTTCCGGAATGGCTTAAGATAAAAAAATGCTTCTTCCTTTTCCTCCATGAAACAGAAGCATCATCTTCATTAACATGCCGTTTCCCAGGGACCCACTCGGAATCCACACTACCACCAAAAGAACCACTCCTGTTAACCACCTCATCATCGCCACTAATCTCCTCGATACCTGAACCACTTATACCACTACTACTACTACTAGTCCCCTTTCCTCCAGCATATCCACTACTACTCGGACTCCCTTGCCCTTCCACCTCAACTTCCACATCCGAATTATTCCTCCACAACACTCCTTCGCCTCTCGAATTTCCAACTACTTCCTTCTCTACTTTTGTATACGAATCCGAACCTAATAGATCCACTTGTCCAGAATTTTCAACTACTTGTCCGACTCGCGAATGTGAATCGGAAGGTAATGAACCGATATTTTCTGGATTTTCAGCTACTGCTACTTGTTGGAGTTGCAAATGTGAATCGGAAGGTAATGTATCGATATTTTGGAGATTTTCAGCTACTGCTGGTTGTTGGAGTTGCGAATGCGAATGGGAAGGCAACGTATCGATAATTTGGGGATGAGGAGGGTGAAATGGGGGTTTTGAGTGGTGGTGATTGAGTGAAATAGAGGTTAATTGACCTTCAATAGCGTCGAGAGATTGGTCAATTGAAGTGGTAGGGTTAGGGTTTTGGTCAATTGAGTCGGTTTGTGATGAGGAGTTGGCATCATCTTCGGGTAACATCGGAGTGAAGAGATATATGTGTATGGTGTTGTATGTATATGCGGAAATGGAGAATTGGGGGTACAGTAACAGGAGTTTTCTTGGTTTGTTATGTTGGGTAGTTGAGGAGGAGAAGAAATATTGTTTTTCCTATCGCACGCTATTTGTTA

**Gene abbreviation**-*TIP41*. **Gene ID**- Unigene27202. **Homolog locus**-AT4G34270. **Length**-1070. **Gene sequence**-TAAAAAGTAAAATACTGTGAAGATATAAATAAAACTTTCCCAGCATGTAATGATAAAATACAGCATTTCCATACTATAAAGGATTAGTATAAGCGCACTGCGTACATGTTAATCAGGAATTATAAGCTTTTGGGTCTTATGCAAAATGATAGGAAGTCTTTGGCTGATGCTGCTTGGATCAATATACGCAGCACAATCAGAAGGATATCCTTTAGAAGCTAGTGCTTGAAATGTGGCTTCTCTCCAGCAACATTCTCTCAGAAGAACTGGTTTTTTACCCTCACCAAAAATGCAATGGATGCGTGTGTCTCTTAAACGCATAAGCACACCATCAACTCTAAGCCAAAAACGCAAGAGAAGAAACCAACAGCTTGGCATCACTCTCACTTTTACTGTTAAAAGCGACACTCCACTATCAGCCAATTCATCTTCATAGAAGATCACCTCATCATAGAAGAGAATAGGCTCTTTTGATGCAAGTGCAGTCAAATTAATTCGTTCCTCGCAGTCCTCCCAACGAAGCTTGCAACTGCCTTCATCAGAGATTGTATCCCTCTCTGAGTTTTTCTCAACAGTTTCACTTCCACAATATGGTGTTGTAAATGTGTAGTCATAATCTAATATCACCTGCTGGAAAGGTTTGCTTCTAAATTTCCATTTTGCTGCTGCAGGGACCTCAACTGGTGGTAATTTTTCCTGCTTCCAACCAACTAGAGAATCAAATGCGTTGAAATGAATCTTTACATCACTCTTCAAGTGCTTGAGAACCAATGAACTTTCCCCAAAAACCATTTCTGGCAAGTGAGAGGTCTGAAGCTTTTGTTCCCACAATTGGACGTTGACTGAGTTGAGGATGGGGAACTTGCGCGTTTCAATCTCCCAACCGTTAATTATTAAACCAGTTCTTCCGTCGGGAAGGAGCTGAGCTCCGGCAGCTTTAAGCTCTTCCTTGTCGATCTCAACTTCCATATTCCGACTTGTTTACCTGATCAGCCGGTCACCAGTTGCCTATATATATCTTCAAGTGACAGCGAGCTCTAATGATTCTTTCTTCCGATTTTCATTTATTC

**Gene abbreviation**-*UBQ10*. **Gene ID**-Unigene1630. **Homolog locus**-AT4G05320. **Length**-1088. **Gene sequence**-ACGTGGTGGCATGCAAATCTTTGTCAAGACCCTAACTGGAAAGACAATCACCTTGGAAGTAGAGAGCTCCGATACGATTGACAATGTGAAGGCAAAAATCCAAGATAAAGAGGGAATTCCTCCAGATCAGCAGAGATTGATCTTTGCTGGCAAGCAGTTGGAGGATGGAAGGACTTTGGCTGATTACAACATTCAGAAGGAGTCTACTCTTCATTTGGTGCTAAGGCTGAGGGGTGGAATGCAGATATTTGTGAAGACTTTGACAGGAAAGACTATTACCTTGGAGGTAGAGAGCTCGGACACCATTGATAATGTCAAAGCAAAAATCCAGGACAAAGAAGGTATCCCGCCAGACCAGCAGAGGCTGATTTTTGCAGGCAAACAGCTTGAGGATGGTCGTACACTTGCAGACTACAATATCCAGAAGGAATCTACCCTCCATTTGGTGCTCCGCCTCAGAGGAGGGATGCAGATCTTTGTCAAGACTTTGACTGGTAAGACCATTACCCTGGAGGTTGAAAGCTCGGACACGATTGATAATGTGAAGGCGAAGATCCAGGACAAGGAGGGGATCCCACCAGATCAGCAGCGGTTGATTTTTGCTGGAAAGCAGCTAGAAGATGGAAGGACCTTGGCCGACTACAACATTCAGAAAGAGTCTACCCTTCACCTCGTTCTTCGTCTCCGTGGTGGTGCATTTTAGGGTTTTGATCCCTGCTTAAACTTTCTGAATTTCTGTTTGATTTTAGTTGAAACTGTATGTGTGCTTGTCTTGAAAGACAATGAATCTAAGTTTTATATTTCCTTGAACGTTTTTATGTGGAATGTTATGTGTTTGTGATTTCAAATTAATGTTCATTTTGCAAAAAAGAATGTGTCAGCTTCGCTGTTCCGAACTGATGGTACAAAATTAGTACTTAAAATTTAGAAAATAGGGTAATAGTGTAGAGGTTCTAATCTGTTGATATTAACATTGGCAATGAGTTTGAAGACACAGAAACACGTTGTTTAGCTCTAAGGACCAGTTTTTTCGAGGCTGAAATCTGTCTAATTTGTTTGTTTTTCCCTGGACAAAATTTATCTTGTTG

**Gene abbreviation**-*ACT*. **Gene ID**- CL614.Contig15. **Homolog locus**-AT3G18780. **Length**-1573. **Gene sequence**-TCCGGGAAAATAAGTATAAAAATGACATTGAGGTAAAGTTCTAAAAAAATTCATAGAATAAGACACATTGACAAATTACTCCTCAATTATAAGCATTGTAGTAACAAGCCACAATTGAGGCCAGAAGATAATAGAAGGCTTGTCATCTTTAGCATACATCCAGAGGACACAAGGACTGATATGTGAACCACAGTGGACAAAAAGATGATACATCAAAATCACATTATCCTCCCAATCAACCACAACATTGAGTTCACATAAAAAAAATACACGAAAATCCAACTAAATTGAAAAAGGAATTCAACCTCAATGCAAATACTATAGAACTTAAAAACACTTCCTGTGCACGATTGATGGACCAGATTCGTCATATTCACCCTTGGATATCCACATCTGTTGGAAGGTGCTGAGAGATGCAAGAATGGATCCTCCGATCCAGACACTGTATTTTCTCTCCGGTGGTGCAACAACTTTGATCTTCATGCTGCTGGGAGCAAGGGCAGTAATTTCCTTGCTCATACGATCTGCAATACCGGGGAACATTGTTGAACCACCACTGAGCACTATGTTTCCATAGAGATCCTTCCTGATATCGACATCACACTTCATGATGGAGTTGTAAGTGGTTTCATGGATTCCAGCAGCTTCCATCCCGATCAGAGATGGCTGGAACAGGACTTCTGGGCAACGGAATCTCTCAGCTCCAATTGTAATAACTTGTCCATCAGGCAATTCATAGTTCTTTTCCACAGAAGAGCTACTCTTTGAGGTTTCAAGCTCTTGCTCGTAGTCAAGAGCAACATAGGCAAGTTTCTCCTTCATGTCACGAACAATTTCCCGCTCAGCAGTGGTGGTGAACATGTAACCTCTCTCCGTTAAGATCTTCATGAGAGAATCAGTGAGATCACGACCAGCAAGGTCGAGACGGAGAATGGCATGGGGAAGGGCATATCCTTCGTAGATTGGTACAGTATGGCTCACACCATCACCAGAATCCAGCACAATACCAGTAGTACGACCACTTGCATATAGAGAAAGAACAGCCTGGATGGCAACATACATGGCAGGAACATTAAACGTCTCAAACATAATCTGAGTCATTTTCTCCCTGTTGGCCTTCGGATTGAGAGGTGCTTCAGTCAAAAGAACTGGGTGCTCCTCAGGAGCAACTCGAAGCTCATTGTAAAAGGTATGATGCCAAATTTTCTCCATGTCATCCCAATTACTAACAATACCGTGCTCAATCGGATATTTCAAGGTAAGAATACCTCTCTTCGATTGGGCTTCATCACCAACATAGGCATCCTTCTGCCCCATCCCGACCATAACACCAGTATGTCTGGGCCTACCAACAATACTGGGGAATACTGCTCTGGGAGCATCATCACCAGCAAAACCAGCCTTCACCATTCCAGTTCCATTGTCACACACAAGGGGTTGGATATCCTCAGCATCGGCCATTATATATCACGATAAATTTTCCGGTGACGACGGCGGCGGCGAGAGTCTCTCTCTAGATATTCTCTCTCTCTCTCTCTAGGTTGTAACGATGAAGGAGGATATGAGCTTTAGG

**Gene abbreviation**-*TUBA*. **Gene ID**-Unigene32860. **Homolog locus**-AT5G12250. **Length**-1821. **Gene sequence**-CAAGAGACTTGCAGATTATCAATCAATCAATCACAAAGTAAACTTTGCTTTTGTAACACAAGCAAGCAAAAACTATGAAATCTGCAAATAAAACTGAATATAATAACAAAGCCAAGTCAAATTCTTAACAAGATTTTGCAAACCGTCTTTGAGAAATTAAACAAACCACATAATAGCTAGACAACAGCTTCAGAAGACTGAATATTGTTCCCAAAAGGCAACAAACAGTTGCAGCATCCTACATACTCAGCAGTTCACACAGTACACTCATGGCAGAACACCTTAGATTTCTTGCCCCTCCTCTTCTTCATCTTCAAAATATTCTCCCTCCTCATCAGCAGTTGCATCCTGGTACTGCTGATACTCAGACACAAGATCGTTCATATTGCTTTCAGCCTCGGTGAATTCCATCTCGTCCATACCTTCGCCTGTGTACCAATGCAAAAAGGCCTTTCTCCTGAACATAGCAGTAAACTGCTCACTCACACGCCTGAACATCTCCTGAATTGAAGTCGAATTCCCAATGAAAGTAGAAGCCATTTTCAAGCCAGTCGGTGGGATATCACAAACAGTTGACTTCACATTGTTTGGGATCCATTCAACAAAGTACGATGAGTTCTTGTTCTGAACATTGATCATCTGCTCATCAACCTCCTTGGTGCTCATCTTTCCTCTGAACACAGCAGAAGCTGTCAAGTATCTACCATGGCGGGGATCAGCTGCGCACATCATGTTCTTGGAATCCCACATCTGTTGGGTAAGCTCAGGCACACTCAAAGCACGATACTGTTGGGAACCACGGGATGTAAGAGGTGCAAAACCCACCATAAAGAAATGAAGCCTAGGGAAGGGGATTAGATTTACAGCCAACTTCCTGAGGTCAGAGTTCAACTGACCAGGGAAACGCAAGCAACATGTAACACCAGACATTGTGGCCGAAATCAAGTGGTTGAGATCACCAAAGCTGGGTGTGGTTAGTTTCAAGGTACGGAAGCAAATGTCATACAGAGCCTCATTGTCAAGAACCATGCACTCATCAGCATTTTCAACAAGTTGATGAACAGAAAGAGTAGCATTATAAGGCTCAACCACAGTATCAGAAACCTTGGGTGATGGGAAAACTGAGAAAGTAAGCATCATACGGTCGGGATACTCCTCTCTGATTTTTGAAATCAGAAGTGTACCCATTCCAGATCCAGTTCCACCACCGAGTGAATGACACACCTGAAACCCTTGAAGACAGTCACAATTCTCAGCTTCCTTCCTCACAACATCAAGCACCGAGTCAATTAACTCAGCACCTTCAGTATAGTGACCTTTGGCCCAATTATTACCAGCACCAGATTGACCAAACACAAAGTTATCAGGCCTGAAGATCTGACCGTAGGGTCCAGATCGGAGACTATCCATAGTACCAGGCTCCAGATCCATAAGCACAGCCCTGGGAACATACCTCTGAGAACTGGCTTCATTGTAATACACATTGATTCGCTCCAATTGAATGTCGGTGTCCCCCTGGTAACGCCCTGTGGAATCGATCCCGTGCTCGGCGCAGATCACTTCCCAGAACTTTGCTCCGATCTGGTTTCCACATTGACCGCCCTGAATGTGAAGAATTTCTCTCATTTTCGATTGGTTTTTAGAGAGAGAAGGTGTTTAGAGAGAGAGTGTGTGTGAAAGTGCGAGTGAGAGATGAAATGAGAGGCGGTGACTGTGGGTAAGGTGTTATTTTTATATGTAAATTGTGTGGGTTTGTGTGAGTGTTTTATAGATACAGGAAAAGGGGTTTTGTTTTTGTGTTGTGTATGTATTGATTTGGAT

Table S2. Ct values of 11 candidate genes of *A. decursiva*.

| **Treatments** | **Replicates** | ***CYP2*** | | ***EXP-1*** | ***GAPDH*** | ***NCBP2*** | ***PP2A*** | ***PTBP*** | ***SAND*** | ***TIP41*** | ***UBQ10*** | ***ACT*** | ***TUBA*** |
| --- | --- | --- | --- | --- | --- | --- | --- | --- | --- | --- | --- | --- | --- |
| UV | 1 | 27.45 | 30.16 | | 27.73 | 16.46 | 22.42 | 27.39 | 24.92 | 26.55 | 22.14 | 21.35 | 29.37 |
|  |  | 27.33 | 27.65 | | 26.24 | 11.21 | 22.5 | 27.52 | 25.48 | 25.48 | 22.25 | 21.44 | 7.86 |
|  |  | 27.19 | 25.28 | | 24.95 | 7.56 | 22.46 | 27.43 | 25.39 | 23.87 | 21.56 | 21.64 | 27.64 |
|  | 2 | 26.59 | 26.65 | | 26.62 | 17.65 | 21.88 | 27.24 | 24.89 | 25.84 | 21.19 | 20.66 | 26.74 |
|  |  | 26.75 | 25.73 | | 24.9 | 10.88 | 22.17 | 27.21 | 24.88 | 24.68 | 21.08 | 21.29 | 27.82 |
|  |  | 27.5 | 25.28 | | 25.31 | 7.82 | 22.53 | 27.34 | 25.24 | 24.31 | 21.16 | 21.61 | 27.63 |
|  | 3 | 27.28 | 28.13 | | 26.47 | 13.93 | 22.66 | 27.31 | 25.6 | 27.26 | 23.8 | 20.95 | 29.66 |
|  |  | 26.71 | 25.44 | | 23.46 | 8.05 | 22.58 | 26.73 | 25.24 | 24.27 | 23.3 | 20.84 | 27.51 |
|  |  | 26.7 | 25.28 | | 23.56 | 8.46 | 22.91 | 27.21 | 25.39 | 24.35 | 23.44 | 20.85 | 27.63 |
| WT | 1 | 27.4 | 28.85 | | 25.34 | 13.99 | 22.35 | 26.8 | 24.27 | 25.73 | 22.32 | 19.25 | 27.74 |
|  |  | 28.27 | 26.3 | | 24.8 | 10.95 | 21.98 | 26.91 | 23.79 | 24.48 | 22.3 | 19.33 | 26.91 |
|  |  | 27.92 | 27.32 | | 22.68 | 8.87 | 22.24 | 26.85 | 23.98 | 22.93 | 21.72 | 19.71 | 25.4 |
|  | 2 | 26.53 | 26.26 | | 26.15 | 16.72 | 22.65 | 27.67 | 24.33 | 25.86 | 21.79 | 18.87 | 23.78 |
|  |  | 27.37 | 25.35 | | 24.69 | 11.25 | 22.65 | 27.4 | 24.48 | 24.29 | 21.41 | 19.58 | 25.59 |
|  |  | 26.67 | 24.43 | | 24.31 | 8.48 | 23.16 | 27.51 | 24.76 | 23.91 | 21.76 | 19.83 | 24.88 |
|  | 3 | 26.23 | 23.78 | | 25.61 | 14.92 | 22.27 | 27.27 | 24.33 | 26.17 | 20.63 | 19.64 | 25.49 |
|  |  | 25.88 | 21.76 | | 22.81 | 8.59 | 21.8 | 26.8 | 23.91 | 23.43 | 20.2 | 19.64 | 24.3 |
|  |  | 25.95 | 21.56 | | 23.27 | 8.76 | 22.24 | 26.92 | 23.77 | 23.22 | 20.21 | 19.62 | 24.48 |
| NaCl | 1 | 30.63 | 27.56 | | 31.63 | 15.97 | 25.79 | 29.99 | 27.84 | 30.15 | 24.96 | 23.81 | 31.42 |
|  |  | 30.72 | 28.56 | | 29.6 | 12.25 | 25.78 | 29.67 | 28.57 | 28.69 | 25.22 | 23.73 | 31.4 |
|  |  | 30.96 | 26.77 | | 28.75 | 8.59 | 26.43 | 30.24 | 28.21 | 27.47 | 24.8 | 24.36 | 28.99 |
|  | 2 | 28.57 | 29.65 | | 28.5 | 14.9 | 25.25 | 29.9 | 26.68 | 28.63 | 24.91 | 22.83 | 25.76 |
|  |  | 30.39 | 27.72 | | 27.36 | 9.9 | 25.7 | 29.32 | 27.37 | 26.91 | 24.92 | 23.33 | 29.86 |
|  |  | 30.67 | 27.48 | | 26.7 | 8.26 | 25.6 | 29.97 | 27.45 | 27.18 | 25.13 | 23.53 | 28.64 |
|  | 3 | 32.47 | 29.49 | | 31.65 | 12.4 | 26.71 | 30.43 | 28.7 | 30.59 | 27.25 | 24.8 | 31.96 |
|  |  | 31.85 | 26.96 | | 28.5 | 8.09 | 25.89 | 30 | 28.5 | 27.34 | 26.22 | 24.53 | 29.72 |
|  |  | 31.71 | 27 | | 28.85 | 8.67 | 26.96 | 30.72 | 29.03 | 27.34 | 26.51 | 24.59 | 29.71 |
| H_2_O_2_ | 1 | 30.87 | 31.42 | | 31.39 | 14.93 | 26.32 | 31.86 | 27.59 | 30.44 | 25.98 | 23.69 | 34.37 |
|  |  | 31.52 | 26.64 | | 28.57 | 8.57 | 26.34 | 30.43 | 28.3 | 28.63 | 26.24 | 24.87 | 29.41 |
|  |  | 30.42 | 26.26 | | 27.64 | 8.46 | 26 | 30.45 | 27.67 | 27.33 | 25.66 | 24.45 | 29.47 |
|  | 2 | 29.37 | 28.64 | | 28.56 | 15.92 | 24.26 | 28.41 | 26.24 | 28.7 | 24.72 | 22.5 | 27.72 |
|  |  | 29.59 | 26.99 | | 27.78 | 10.86 | 24.34 | 28.5 | 25.86 | 26.64 | 24.99 | 22.39 | 28.19 |
|  |  | 30.72 | 26.57 | | 26.89 | 8.51 | 24.55 | 28.59 | 26.76 | 26.63 | 25.19 | 22.97 | 28.24 |
|  | 3 | 33.47 | 32.63 | | 32 | 7.99 | 27.6 | 32.54 | 29.58 | 31.43 | 28.88 | 25.64 | 33.28 |
|  |  | 32.31 | 30.25 | | 29.25 | 8.22 | 27.26 | 31.88 | 28.79 | 28.44 | 27.91 | 25.77 | 31.16 |
|  |  | 32.18 | 29.41 | | 29.35 | 8.51 | 27.68 | 31.35 | 28.94 | 28.47 | 28.63 | 25.65 | 31.27 |
| PEG | 1 | 29.39 | 26.89 | | 29.33 | 9.81 | 23.96 | 28.32 | 26.27 | 28.87 | 24.86 | 21.99 | 29.78 |
|  |  | 29.84 | 28.6 | | 26.99 | 10.93 | 23.91 | 28.77 | 26.45 | 27.44 | 24.39 | 22.53 | 28.21 |
|  |  | 36.56 | 26.54 | | 26 | 8.23 | 24.33 | 28.8 | 26.35 | 25.45 | 23.88 | 22.54 | 27.86 |
|  | 2 | 26.67 | 25.96 | | 25.79 | 15.9 | 22.66 | 27.38 | 23.97 | 26.45 | 20.96 | 19.64 | 24.37 |
|  |  | 26.55 | 25.48 | | 24.75 | 7.81 | 22.43 | 26.73 | 24.34 | 23.84 | 21.93 | 20.33 | 26.51 |
|  |  | 27.52 | 24.95 | | 24.53 | 8.34 | 22.94 | 27.46 | 24.5 | 23.98 | 21.96 | 20.7 | 25.91 |
|  | 3 | 30.44 | 29.67 | | 27.75 | 13.75 | 24.33 | 28.47 | 25.86 | 27.39 | 23.78 | 22.86 | 30.49 |
|  |  | 30.57 | 26.88 | | 25.43 | 8.71 | 23.89 | 30.4 | 25.95 | 25.47 | 23.5 | 22.66 | 29.57 |
|  |  | 29.78 | 26.57 | | 25.63 | 8.98 | 24.37 | 27.63 | 25.63 | 25.27 | 23.45 | 22.91 | 29.4 |
| CuSO_4_ | 1 | 27.66 | 27.58 | | 26.91 | 11.6 | 22.54 | 26.96 | 24.41 | 25.5 | 23.49 | 20.41 | 29.3 |
|  |  | 27.76 | 24.83 | | 25.33 | 10.39 | 22.85 | 27.69 | 24.95 | 24.65 | 23.09 | 20.7 | 26.88 |
|  |  | 27.88 | 23.94 | | 24.4 | 8.61 | 23.2 | 27.64 | 24.67 | 23.51 | 22.51 | 20.94 | 27 |
|  | 2 | 26.62 | 26.39 | | 25.6 | 14.49 | 22.41 | 26.71 | 23.92 | 25.48 | 23.42 | 19.32 | 22.8 |
|  |  | 27.32 | 24.95 | | 23.93 | 7.53 | 22.2 | 26.73 | 24.45 | 23.56 | 23.6 | 19.57 | 29.87 |
|  |  | 27.68 | 25.98 | | 24.29 | 8.32 | 22.73 | 27.52 | 24.73 | 23.65 | 23.57 | 19.93 | 25.01 |
|  | 3 | 27.57 | 24.65 | | 26.47 | 12.94 | 23.7 | 28.45 | 24.73 | 23.94 | 22.91 | 20.86 | 27.86 |
|  |  | 27.26 | 23.89 | | 24.46 | 8.87 | 23.45 | 28.28 | 24.77 | 23.8 | 22.27 | 20.71 | 26.57 |
|  |  | 28.04 | 23.73 | | 25.46 | 8.38 | 23.61 | 29.66 | 25.16 | 22.75 | 22.63 | 20.91 | 26.74 |
| MeJA | 1 | 29.66 | 30.99 | | 28.28 | 8.8 | 24.68 | 28.96 | 27.46 | 28.78 | 25.69 | 22 | 28.57 |
|  |  | 30.19 | 27.89 | | 26.67 | 7.01 | 24.89 | 29.42 | 26.34 | 27.52 | 25.71 | 22.32 | 27.88 |
|  |  | 29.99 | 26.34 | | 25.91 | 8.46 | 25.22 | 29.22 | 26.85 | 25.86 | 25.08 | 22.65 | 26.84 |
|  | 2 | 26.68 | 24.35 | | 25.64 | 15.71 | 22.41 | 27.46 | 24.39 | 25.76 | 23.6 | 19.67 | 26.74 |
|  |  | 27.38 | 24.74 | | 23.96 | 7.5 | 22.38 | 26.8 | 24.45 | 24.22 | 23.86 | 19.9 | 24.95 |
|  |  | 27.93 | 25.43 | | 24.25 | 8.44 | 22.95 | 27.4 | 24.86 | 23.91 | 24.28 | 20.63 | 25.57 |
|  | 3 | 32.71 | 30.52 | | 30.39 | 12.54 | 27.5 | 30.62 | 28.36 | 28.73 | 28.72 | 24.65 | 31 |
|  |  | 32.65 | 29.25 | | 27.96 | 8.54 | 27.39 | 30.93 | 27.92 | 28.46 | 28.2 | 24.58 | 29.74 |
|  |  | 33.49 | 29.74 | | 28.4 | 8.43 | 27.51 | 30.5 | 27.65 | 28.33 | 28.3 | 24.84 | 30.03 |
| Cold | 1 | 28.64 | 30.55 | | 26.76 | 6.88 | 22.31 | 26.21 | 24.39 | 25.91 | 24.38 | 20.35 | 26.93 |
|  |  | 28.34 | 26.63 | | 24.64 | 10.3 | 22.47 | 27.18 | 24.48 | 25.22 | 24.4 | 20.58 | 26.45 |
|  |  | 28.59 | 25.99 | | 23.41 | 6.94 | 22.71 | 27.25 | 24.14 | 23.54 | 24.61 | 20.78 | 26.45 |
|  | 2 | 27.27 | 26.54 | | 24.73 | 13.86 | 21.93 | 26.2 | 23.67 | 24.72 | 22.47 | 20.28 | 26.62 |
|  |  | 28.12 | 24.9 | | 23.91 | 7.33 | 22.2 | 26.3 | 23.82 | 22.78 | 23.97 | 20.52 | 26.99 |
|  |  | 29.05 | 24.86 | | 23.6 | 8.13 | 22.75 | 27.42 | 24.38 | 22.97 | 22.85 | 20.9 | 26.69 |
|  | 3 | 30.35 | 27.63 | | 26.94 | 7.97 | 24.88 | 28.66 | 26.8 | 25.87 | 27.61 | 22.54 | 28.36 |
|  |  | 29.57 | 27.99 | | 25.24 | 8.35 | 24.95 | 29.13 | 26.45 | 36.44 | 26.58 | 22.38 | 27.93 |
|  |  | 30.26 | 28.24 | | 25.21 | 8.91 | 25.27 | 28.86 | 26.45 | 25.77 | 27.07 | 22.62 | 28.36 |

Table S3. Pairwise variation (V _n/n+1_) analysis of nine candidate reference genes calculated using geNorm

|  | NaCl | PEG | Cold | MeJA | UV | H_~~2~~_O_2_ | CuSO_4_ | WT | Total |
| --- | --- | --- | --- | --- | --- | --- | --- | --- | --- |
| V2/3 | 0.117 | 0.171 | 0.111 | 0.158 | 0.085 | 0.106 | 0.131 | 0.074 | 0.176 |
| V3/4 | 0.110 | 0.143 | 0.087 | 0.126 | 0.085 | 0.126 | 0.092 | 0.114 | 0.152 |
| V4/5 | 0.117 | 0.170 | 0.092 | 0.150 | 0.062 | 0.107 | 0.125 | 0.166 | 0.228 |
| V5/6 | 0.107 | 0.183 | 0.086 | 0.128 | 0.178 | 0.119 | 0.158 | 0.134 | 0.187 |
| V6/7 | 0.198 | 0.174 | 0.134 | 0.110 | 0.164 | 0.185 | 0.148 | 0.166 | 0.193 |
| V7/8 | 0.158 | 0.149 | 0.154 | 0.118 | 0.167 | 0.142 | 0.139 | 0.130 | 0.199 |
| V8/9 | 0.148 | 0.146 | 0.179 | 0.104 | 0.155 | 0.158 | 0.151 | 0.128 | 0.173 |
| V9/10 | 0.151 | 0.258 | 0.269 | 0.121 | 0.354 | 0.145 | 0.211 | 0.210 | 0.263 |
| V10/11 | 0.267 | 0.278 | 0.332 | 0.319 | 0.624 | 0.337 | 0.216 | 0.253 | 0.316 |

Table S4. Expression stability values of 11 candidate genes calculated using the Delta Ct method in *A. decursiva*

| **Rank** | **WT** | **Cold** | **MeJA** | **PEG** | **NaCl** | **CuSO4** | **UV** | **H2O2** | **Total** |
| --- | --- | --- | --- | --- | --- | --- | --- | --- | --- |
| **1** | *SAND* | *TUBA* | *SAND* | *SAND* | *ACT* | *SAND* | *CYP2* | *PP2A* | *SAND* |
|  | 1.16 | 1.10 | 1.21 | 1.29 | 1.15 | 1.11 | 1.72 | 1.25 | 1.52 |
| **2** | *PEBP* | *PP2A* | *ACT* | *PP2A* | *PP2A* | *CYP2* | *PTBP* | *PTBP* | *PP2A* |
|  | 1.19 | 1.10 | 1.22 | 1.35 | 1.18 | 1.11 | 1.72 | 1.26 | 1.52 |
| **3** | *PP2A* | *UBQ10* | *PP2A* | *UBQ10* | *SAND* | *PP2A* | *SAND* | *SAND* | *ACT* |
|  | 1.19 | 1.11 | 1.27 | 1.36 | 1.19 | 1.16 | 1.76 | 1.26 | 1.58 |
| **4** | *UBQ10* | *ACT* | *TUBA* | *ACT* | *PTBP* | *ACT* | *PP2A* | *CYP2* | *PTBP* |
|  | 1.21 | 1.12 | 1.30 | 1.38 | 1.22 | 1.16 | 1.77 | 1.33 | 1.59 |
| **5** | *CYP2* | *PTBP* | *PTBP* | *EXP-1* | *UBQ10* | *UBQ10* | *ACT* | *GAPDH* | *GAPDH* |
|  | 1.33 | 1.15 | 1.32 | 1.55 | 1.24 | 1.23 | 1.81 | 1.38 | 1.80 |
| **6** | *ACT* | *SAND* | *CYP2* | *PTBP* | *CYP2* | *GAPDH* | *TIP41* | *UBQ10* | *UBQ10* |
|  | 1.35 | 1.17 | 1.33 | 1.56 | 1.36 | 1.30 | 1.90 | 1.39 | 1.84 |
| **7** | *GAPDH* | *GAPDH* | *UBQ10* | *GAPDH* | *TIP41* | *TIP41* | *GAPDH* | *ACT* | *CYP2* |
|  | 1.37 | 1.22 | 1.62 | 1.60 | 1.43 | 1.36 | 2.07 | 1.40 | 1.88 |
| **8** | *TIP41* | *TIP41* | *GAPDH* | *TIP41* | *GAPDH* | *PTBP* | *UBQ10* | *TIP41* | *EXP-1* |
|  | 1.38 | 1.29 | 1.66 | 1369.00 | 1.57 | 1.42 | 2.15 | 1.44 | 2.05 |
| **9** | *TUBA* | *CYP2* | *EXP-1* | *TUBA* | *EXP-1* | *EXP-1* | *EXP-1* | *TUBA* | *TIP41* |
|  | 1.55 | 1.35 | 2.03 | 1.85 | 1.60 | 1.62 | 2.21 | 1.75 | 2.08 |
| **10** | *EXP-1* | *EXP-1* | *NCBP2* | *CYP2* | *TUBA* | *TUBA* | *NCBP2* | *EXP-1* | *TUBA* |
|  | 2.29 | 1.62 | 2.97 | 2.88 | 1.86 | 2.38 | 3.99 | 1.79 | 2.97 |
| **11** | *NCBP2* | *NCBP2* | *TIP41* | *NCBP2* | *NCBP2* | *NCBP2* | *TUBA* | *NCBP2* | *NCBP2* |
|  | 2.90 | 3.56 | 3.79 | 3.19 | 3.02 | 2.49 | 6.98 | 3.79 | 3.66 |

Table S5. Expression stability values of 11 candidate genes calculated using the RefFinder in *A. decursiva*

| **Rank** | **WT** | **Cold** | **MeJA** | **PEG** | **NaCl** | **CuSO_4_** | **UV** | **H_2_O_2_** | **Total** |
| --- | --- | --- | --- | --- | --- | --- | --- | --- | --- |
| **1** | *SAND* | *SAND* | *TUBA* | *SAND* | *PP2A* | *SAND* | *PTBP* | *SAND* | *SAND* |
|  | 1.41 | 1.57 | 2.55 | 1.19 | 1.68 | 1.19 | 1.86 | 1.96 | 1.19 |
| **2** | *PP2A* | *ACT* | *PTBP* | *PP2A* | *ACT* | *CYP2* | *CYP2* | *PP2A* | *PP2A* |
|  | 2.71 | 2.45 | 2.66 | 1.41 | 1.73 | 1.41 | 1.97 | 2.45 | 1.86 |
| **3** | *UBQ10* | *TUBA* | *PP2A* | *UBQ10* | *PTBP* | *UBQ10* | *ACT* | *PTBP* | *PTBP* |
|  | 3.16 | 2.91 | 3.03 | 3.83 | 2.00 | 4.05 | 3.31 | 3.60 | 2.63 |
| **4** | *PTBP* | *PP2A* | *ACT* | *ACT* | *SAND* | *PP2A* | *PP2A* | *CYP2* | *ACT* |
|  | 3.31 | 3.03 | 3.03 | 3.94 | 3.46 | 4.12 | 3.56 | 3.72 | 3.46 |
| **5** | *ACT* | *CYP2* | *SAND* | *PTBP* | *UBQ10* | *ACT* | *SAND* | *GAPDH* | *GAPDH* |
|  | 3.72 | 4.36 | 3.83 | 4.82 | 5.23 | 4.16 | 3.66 | 3.81 | 5.92 |
| **6** | *CYP2* | *PTBP* | *UBQ10* | *EXP-1* | *CYP2* | *GAPDH* | *TIP41* | *ACT* | *UBQ10* |
|  | 5.96 | 4.47 | 3.94 | 4.95 | 6.45 | 6.05 | 4.14 | 4.09 | 6.00 |
| **7** | *GAPDH* | *GAPDH* | *GAPDH* | *GAPDH* | *TIP41* | *PTBP* | *UBQ10* | *TIP41* | *CYP2* |
|  | 6.03 | 6.88 | 6.51 | 7.00 | 6.88 | 6.62 | 6.93 | 5.26 | 6.65 |
| **8** | *TIP41* | *UBQ10* | *CYP2* | *TIP41* | *EXP-1* | *TIP41* | *GAPDH* | *UBA10* | *EXP-1* |
|  | 6.73 | 7.71 | 6.84 | 8.00 | 7.94 | 7.24 | 7.48 | 6.40 | 7.11 |
| **9** | *TUBA* | *EXP-1* | *TIP41* | *TUBA* | *GAPDH* | *EXP-1* | *EXP-1* | *TUBA* | *TIP41* |
|  | 8.45 | 8.74 | 7.33 | 9.00 | 8.21 | 9.00 | 9.00 | 9.24 | 9.00 |
| **10** | *EXP-1* | *NCBP2* | *EXP-1* | *CYP2* | *TUBA* | *TUBA* | *NCBP2* | *EXP-1* | *TUBA* |
|  | 10.00 | 10.00 | 10.24 | 10.00 | 10.00 | 10.00 | 10.00 | 9.74 | 10.00 |
| **11** | *NCBP2* | *TIP41* | *NCBP2* | *NCBP2* | *NCBP2* | *NCBP2* | *TUBA* | *NCBP2* | *NCBP2* |
|  | 11.00 | 11.00 | 10.74 | 11.00 | 11.00 | 11.00 | 11.00 | 11.00 | 11.00 |

**Table S6.** Rankings of 11 candidate reference genes based on stability values calculated using different type of software.

| **Rank** | **1** | **2** | **3** | **4** | **5** | **6** | **7** | **8** | **9** | **10** | **11** |
| --- | --- | --- | --- | --- | --- | --- | --- | --- | --- | --- | --- |
| **WT** | | | | | | | | | | | |
| **geNorm** | *PP2A \|* | *SAND* | *PTBP* | *ACT* | *UBQ10* | *CYP2* | *GAPDH* | *TIP41* | *TUBA* | *EXP-1* | *NCBP2* |
| **NormFinder** | *UBQ10* | *SAND* | *GAPDH* | *TIP41* | *PTBP* | *PP2A* | *CYP2* | *ACT* | *TUBA* | *EXP-1* | *NCBP2* |
| **BestKeeper** | *ACT* | *SAND* | *PP2A* | *PTBP* | *UBQ10* | *CYP2* | *TUBA* | *TIP41* | *GAPDH* | *EXP-1* | *NCBP2* |
| **Comprehensive Ranking** | *SAND* | *PP2A* | *UBQ10* | *PTBP* | *ACT* | *CYP2* | *GAPDH* | *TIP41* | *TUBA* | *EXP-1* | *NCBP2* |
| **Cold** | | | | | | | | | | | |
| **geNorm** | *PP2A \|* | *SAND* | *ACT* | *PTBP* | *CYP2* | *TUBA* | *UBQ10* | *GAPDH* | *EXP-1* | *NCBP2* | *TIP41* |
| **NormFinder** | *SAND* | *ACT* | *TUBA* | *PP2A* | *PTBP* | *CYP2* | *GAPDH* | *UBQ10* | *EXP-1* | *NCBP2* | *TIP41* |
| **BestKeeper** | *TUBA* | *CYP2* | *ACT* | *PTBP* | *GAPDH* | *SAND* | *PP2A* | *EXP-1* | *UBQ10* | *NCBP2* | *TIP41* |
| **Comprehensive Ranking** | *SAND* | *ACT* | *TUBA* | *PP2A* | *CYP2* | *PTBP* | *GAPDH* | *UBQ10* | *EXP-1* | *NCBP2* | *TIP41* |
| **MeJA** | | | | | | | | | | | |
| **geNorm** | *PP2A \|* | *ACT* | *CYP2* | *UBQ10* | *PTBP* | *SAND* | *TUBA* | *GAPDH* | *TIP41* | *EXP-1* | *NCBP2* |
| **NormFinder** | *TUBA* | *PTBP* | *SAND* | *GAPDH* | *UBQ10* | *PP2A* | *ACT* | *TIP41* | *CYP2* | *EXP-1* | *NCBP2* |
| **BestKeeper** | *PTBP* | *SAND* | *ACT* | *UBQ10* | *TIP41* | *TUBA* | *PP2A* | *GAPDH* | *CYP2* | *NCBP2* | *EXP-1* |
| **Comprehensive Ranking** | *TUBA* | *PTBP* | *PP2A* | *ACT* | *SAND* | *UBQ10* | *GAPDH* | *CYP2* | *TIP41* | *EXP-1* | *NCBP2* |
| **PEG** | | | | | | | | | | | |
| **geNorm** | *PP2A \|* | *SAND* | *ACT* | *UBQ10* | *PTBP* | *EXP-1* | *GAPDH* | *TIP41* | *TUBA* | *CYP2* | *NCBP2* |
| **NormFinder** | *SAND* | *PP2A* | *UBQ10* | *ACT* | *EXP-1* | *PTBP* | *GAPDH* | *TIP41* | *TUBA* | *CYP2* | *NCBP2* |
| **BestKeeper** | *PP2A* | *SAND* | *PTBP* | *EXP-1* | *ACT* | *UBQ10* | *GAPDH* | *TIP41* | *TUBA* | *CYP2* | *NCBP2* |
| **Comprehensive Ranking** | *SAND* | *PP2A* | *UBQ10* | *ACT* | *PTBP* | *EXP-1* | *GAPDH* | *TIP41* | *TUBA* | *CYP2* | *NCBP2* |
| **NaCl** | | | | | | | | | | | |
| **geNorm** | *PP2A \|* | *ACT* | *SAND* | *PTBP* | *UBQ10* | *CYP2* | *EXP-1* | *TIP41* | *GAPDH* | *TUBA* | *NCBP2* |
| **NormFinder** | *PTBP* | *PP2A* | *ACT* | *SAND* | *TIP41* | *UBQ10* | *GAPDH* | *CYP2* | *EXP-1* | *TUBA* | *NCBP2* |
| **BestKeeper** | *PTBP* | *PP2A* | *ACT* | *SAND* | *UBQ10* | *CYP2* | *EXP-1* | *TIP41* | *GAPDH* | *TUBA* | *NCBP2* |
| **Comprehensive Ranking** | *PP2A* | *ACT* | *PTBP* | *SAND* | *UBQ10* | *CYP2* | *TIP41* | *EXP-1* | *GAPDH* | *TUBA* | *NCBP2* |
| **CuSO_4_** | | | | | | | | | | | |
| **geNorm** | *CYP2 \|* | *SAND* | *ACT* | *PP2A* | *PTBP* | *UBQ10* | *GAPDH* | *TIP41* | *EXP-1* | *TUBA* | *NCBP2* |
| **NormFinder** | *CYP2* | *SAND* | *UBQ10* | *GAPDH* | *ACT* | *PP2A* | *TIP41* | *PTBP* | *EXP-1* | *TUBA* | *NCBP2* |
| **BestKeeper** | *SAND* | *CYP2* | *UBQ10* | *PP2A* | *ACT* | *PTBP* | *TIP41* | *GAPDH* | *EXP-1* | *TUBA* | *NCBP2* |
| **Comprehensive Ranking** | *SAND* | *CYP2* | *UBQ10* | *PP2A* | *ACT* | *GAPDH* | *PTBP* | *TIP41* | *EXP-1* | *TUBA* | *NCBP2* |
| **H_2_O_2_** | | | | | | | | | | | |
| **geNorm** | *SAND \|* | *ACT* | *PP2A* | *CYP2* | *UBQ10* | *PTBP* | *GAPDH* | *TIP41* | *TUBA* | *EXP-1* | *NCBP2* |
| **NormFinder** | *GAPDH* | *PTBP* | *TIP41* | *PP2A* | *SAND* | *CYP2* | *UBQ10* | *ACT* | *TUBA* | *EXP-1* | *NCBP2* |
| **BestKeeper** | *SAND* | *CYP2* | *PP2A* | *TIP41* | *ACT* | *GAPDH* | *PTBP* | *UBQ10* | *EXP-1* | *TUBA* | *NCBP2* |
| **Comprehensive Ranking** | *SAND* | *PP2A* | *PTBP* | *CYP2* | *GAPDH* | *ACT* | *TIP41* | *UBQ10* | *TUBA* | *EXP-1* | *NCBP2* |
| **UV** | | | | | | | | | | | |
| **geNorm** | *CYP2 \|* | *ACT* | *PTBP* | *SAND* | *PP2A* | *UBQ10* | *TIP41* | *GAPDH* | *EXP-1* | *NCBP2* | *TUBA* |
| **NormFinder** | *TIP41* | *PTBP* | *CYP2* | *PP2A* | *SAND* | *ACT* | *GAPDH* | *UBQ10* | *EXP-1* | *NCBP2* | *TUBA* |
| **BestKeeper** | *PTBP* | *PP2A* | *SAND* | *ACT* | *CYP2* | *UBQ10* | *TIP41* | *GAPDH* | *EXP-1* | *NCBP2* | *TUBA* |
| **Comprehensive Ranking** | *PTBP* | *CYP2* | *ACT* | *PP2A* | *SAND* | *TIP41* | *UBQ10* | *GAPDH* | *EXP-1* | *NCBP2* | *TUBA* |
| **Total** | | | | | | | | | | | |
| **geNorm** | *PP2A \|* | *SAND* | *ACT* | *PTBP* | *CYP2* | *UBQ10* | *GAPDH* | *EXP-1* | *TIP41* | *TUBA* | *NCBP2* |
| **NormFinder** | *SAND* | *PP2A* | *PTBP* | *ACT* | *GAPDH* | *UBQ10* | *CYP2* | *EXP-1* | *TIP41* | *TUBA* | *NCBP2* |
| **BestKeeper** | *PTBP* | *SAND* | *PP2A* | *ACT* | *EXP-1* | *UBQ10* | *GAPDH* | *CYP2* | *TIP41* | *TUBA* | *NCBP2* |
| **Comprehensive Ranking** | *SAND* | *PP2A* | *PTBP* | *ACT* | *GAPDH* | *UBQ10* | *CYP2* | *EXP-1* | *TIP41* | *TUBA* | *NCBP2* |
